# Transcripts with *in silico* predicted RNA structure are enriched everywhere in the mouse brain

**DOI:** 10.1186/1471-2164-13-214

**Published:** 2012-05-31

**Authors:** Stefan E Seemann, Susan M Sunkin, Michael J Hawrylycz, Walter L Ruzzo, Jan Gorodkin

**Affiliations:** 1 Center for non-coding RNA in Technology and Health, University of Copenhagen, Denmark; 2, IBHV, University of Copenhagen, Denmark; 3 Allen Institute for Brain Science, , USA; 4 Computer Science and Engineering and Genome Sciences, Univ. of Washington, USA; 5 Fred Hutchinson Cancer Research Center, , USA

## Abstract

**Background:**

Post-transcriptional control of gene expression is mostly conducted by specific elements in untranslated regions (UTRs) of mRNAs, in collaboration with specific binding proteins and RNAs. In several well characterized cases, these RNA elements are known to form stable secondary structures. RNA secondary structures also may have major functional implications for long noncoding RNAs (lncRNAs). Recent transcriptional data has indicated the importance of lncRNAs in brain development and function. However, no methodical efforts to investigate this have been undertaken. Here, we aim to systematically analyze the potential for RNA structure in brain-expressed transcripts.

**Results:**

By comprehensive spatial expression analysis of the adult mouse *in situ* hybridization data of the Allen Mouse Brain Atlas, we show that transcripts (coding as well as non-coding) associated with *in silico* predicted structured probes are highly and significantly enriched in almost all analyzed brain regions. Functional implications of these RNA structures and their role in the brain are discussed in detail along with specific examples. We observe that mRNAs with a structure prediction in their UTRs are enriched for binding, transport and localization gene ontology categories. In addition, after manual examination we observe agreement between RNA binding protein interaction sites near the 3’ UTR structures and correlated expression patterns.

**Conclusions:**

Our results show a potential use for RNA structures in expressed coding as well as noncoding transcripts in the adult mouse brain, and describe the role of structured RNAs in the context of intracellular signaling pathways and regulatory networks. Based on this data we hypothesize that RNA structure is widely involved in transcriptional and translational regulatory mechanisms in the brain and ultimately plays a role in brain function.

## Background

In neurons, RNA molecules often have to travel long distances between transcriptional origin (nucleus) and functional destination (axon, synapses, dendrites). Dendrites contain thousands of postsynaptic sites and long-lasting forms of activity-dependent synaptic modifications (memory storage) are believed to require local protein synthesis. Local protein translation implies that mRNAs are transported from the nucleus and localized to dendrites and synapses [1,2]. It has been speculated that RNA secondary structures in mRNA untranslated regions (UTRs) are involved in these processes [3,4]. In addition, numerous noncoding RNAs (ncRNAs) are expressed in brain [5,6] and mounting evidence indicates important contributions of ncRNAs in brain functions such as memory formation and maintenance [7,8], as well as a host of other functions in mammalian cells. This study further explores these connections by combining the large scale *in situ* hybridization data of the Allen Mouse Brain Atlas (http://mouse.brain-map.org) [9] with in silico predictions of conserved RNA secondary structure, revealing extensive enrichment of such structures in the adult mouse brain transcriptome.

Post-transcriptional regulation of RNA splicing, editing, transport, stability, localization and translation through UTR signals plays an important role in controlling gene expression. Important examples of stable RNA secondary structures are known in both 5’ UTRs [10] and 3’ UTRs. For instance, the 84-nucleotide (nt) long structure-anchored repression element (CAESAR) in *CTGF*[4] is highly conserved in structure but not in sequence, and is suspected to inhibit translation and affect mRNA stability [11]. Other structural mRNA elements, such as the selenocysteine insertion sequence (SECIS) element and nanos 3’ UTR TCE, are targets of RNA binding proteins. Stem-loop structures in untranslated regions are sometimes critical for proper mRNA localization [12-14], such as translocation of the *MAPT* mRNA along axonal microtubules [15] and *ASH1* mRNA to the cortical actin cytoskeleton [16]. RNA binding proteins might localize the *RAB1A* mRNA to specific cytoplasmic regions through recognition of its highly conserved 3’ UTR sequence and structure, so that translation would occur close to the location of the respective protein regulating intracellular vesicle transport [17]. A predicted stable RNA structure overlaps the RNA localization region in the 3’ UTR of the mRNA encoding myelin basic protein MBP. The structure (but not the sequence) is conserved in human, mouse and rat [18]. The highest affinity site of the RNA-binding protein *Qk1* is located within the RNA localization region of *MBP*, suggesting a possible role for *Qk1* in restricting *MBP* mRNA to the myelin membrane [19]. In a very distinct manner, many 3’ UTRs in mouse are reported to be expressed separately from their mRNAs in a developmentally regulated manner [20], and some reported regulatory mutations in 3’ UTRs do not appear to act *in cis* to regulate the expression of the associated mRNA. Some structured 3’ UTRs may, thus, act *in trans* as ncRNAs [21].

Long noncoding RNAs (lncRNAs) have recently received increased attention due to their functional diversity in basic molecular and cellular biology [21-24]. In particular, they appear to be deeply entwined with cellular regulatory machinery, both as targets of important transcription factors [25], and as direct cis- and trans-regulators of gene expression through interactions with transcription factors or as indirect regulators through an RNA-binding protein intermediate (transcription factor co-regulators) [26]. Furthermore, they have demonstrated roles in regulation of dosage compensation, imprinting, chromatin state, and epigenetic inheritance by DNA methylation [26]. A hallmark of many small ncRNAs is the critical role of RNA secondary (and tertiary) structure. RNA structure also may have major functional implications for lncRNAs as shown, *e.g.*, for the noncoding co-factor MEG3 of the tumor repressor *p53*[27], and the *p53* regulated transcriptional repressor *lincRNA-p21*, which is tethered to *hnRNP-K* for its proper localization [28].

Several genome-scale screens for stable, conserved RNA secondary structures have found known RNA families and many potentially novel ncRNAs ( [29], [30], [31], [32]), albeit with significant false discovery rates [33,34]. Classical transfer RNAs, ribosomal RNAs, some microRNAs and many other functional ncRNAs have a weakly conserved sequence, and instead, have a highly conserved functional secondary structure. Hence, comparative analyses that focus on sequence conservation and ignore potential conservation of secondary structure underestimate ncRNA prevalence. Here, we apply to search for RNA secondary structures. The method attempts to create structurally optimal alignments from unaligned orthologous input sequences using an expectation-maximization algorithm. Both thermodynamic energies and evidence for conservation of secondary structure, *e.g.* presence of compensatory mutations in putative helices, are part of the evaluation criteria. An appropriate background model distinguishes between significant RNA structures, *e.g.* putative ncRNAs, and structured background.

The key question addressed in this paper is the extent to which RNA structures, both in noncoding transcripts and the UTRs of protein coding transcripts, play biologically important roles in the brain. We address this question by analyzing transcripts expressed in the adult mouse brain as cataloged in the Allen Mouse Brain Atlas (Atlas) for their potential to contain RNA structures predicted by . There are Atlas probes for approximately 20,000 RNA transcripts in the adult mouse brain, visualized at cellular resolution by in situ hybridization (ISH) [9]. Of these transcripts, 16,900 exhibit cellular expression above background in the adult mouse brain [9]. Expression data within the ISH images is identified and mapped to defined regions [35]. This mapped expression data can be used to examine global and spatial expression patterns and to find genes with similar spatial expression profiles. Although the majority of Atlas transcripts represent protein coding genes, Mercer *et al.*[36] identified well over 1,000 Atlas riboprobes as putative lncRNAs and affirmed the expression patterns of some previously described lncRNAs, such as *Evf Gtl2Gomafu*, and *Sox2ot*.

## Results

### Structured Allen Mouse Brain Atlas riboprobes

Table 1 shows a summary of the Allen Mouse Brain Atlas riboprobes used in this study. For this study, we exclusively consider probes whose expression is above ISH background in the adult mouse brain [9]. Our main concern is whether “structured probes”, i.e., those containing a conserved RNA secondary structure as predicted by , are in any other way a distinct population compared to “unstructured probes”, i.e., those lacking such predictions. Structured probes are further categorized into (1) putative ncRNAs (or simply ncRNAs) and (2) UTRs. A probe is classified as an ncRNA (of which some presumably are lncRNAs) if the entire probe is intergenic or intronic without protein coding potential; it is classified as UTR if the probe overlaps an annotated UTR. Probes in coding exons are not analyzed.

**Table 1:** Structured Allen Mouse Brain Atlas riboprobes

	Only	Only	Overlap	Overlap	
	Intergenic	Intronic	5’ UTR	3’ UTR	Total
Expressed structured ncRNAs	141	10			151
Expressed structured UTRs			817	4,502	5,126
Expressed Atlas probes	462	15	2,492	8,108	16,483

Structured Allen Mouse Brain Atlas riboprobes have RNA secondary structures predicted by , are expressed above background in the adult mouse brain and are mapped to the mouse genome (mm8). We subdivided structured probes into two genomic locations: (1) structured (long) noncoding RNAs (ncRNAs) have no protein coding potential and the entire probe is intergenic or intronic, and (2) structured UTRs are probes overlapping a UTR exon annotated in UCSC or RefSeq gene tracks with an RNA structure prediction. Total numbers of expressed Allen Mouse Brain Atlas probes (structured and non-structured) are listed. Detailed information about probes and structures are contained in Additional file 1.

**Figure 1:**
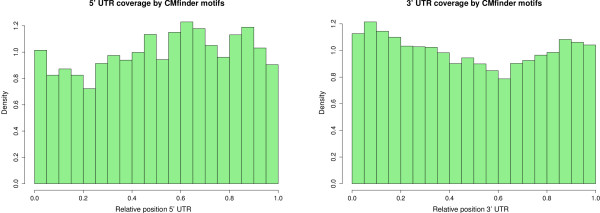
**Relative location of predicted UTR structures.** Relative location of predicted structures in UTRs annotated in UCSC known genes that overlap the Allen Mouse Brain Atlas probes. Using we predicted 1,367 structured loci (average length 76 nt) in 5’ UTRs (average length 284 nt) of 817 expressed mRNAs and 11,551 structured loci (average length 82 nt) in 3’ UTRs (average length 946 nt) of 4,502 expressed mRNAs. X-axis equal 0 describes the 5’ end of UTRs.

By considering all annotated UTRs of full-length transcripts we find many additional structures, often at the end of longer alternative UTRs [37]. However, the expression of these variants in the brain is unknown, which is why we consider only those portions of UTRs that are overlapped by Atlas probes. For instance, one isoform of mouse VEGF-related factor gene (Vegfb) lacking a 3’UTR is expressed in brain [38]. We predict a RNA structure in one alternative 3’UTR of Vegfb, but we annotate it as a non-structured UTR because the Atlas probe does not overlap the extended 3’ UTR structure.

 predicts conserved RNA structures in the genomic context of 11,998 Atlas riboprobes of which 10,516 probes are expressed above background (see Methods for mapping criteria). The amount of predicted structures overlapping expressed probes is sensitive to GC content but significantly larger in all GC bins than expected by chance (see Additional file 2: Table S1). We mapped the expressed structured probes to UCSC [39] and RefSeq [40] gene tracks and obtained 5,126 probes with predicted structures in annotated untranslated regions (817 and 4,502 probes in 5’ UTR and 3’ UTR regions, respectively). The predicted structures are enriched at the flanks of UTRs, see Figure 1. In contrast, 4,467 expressed Atlas probes map to protein coding genes lacking structure predictions in their UTRs. Riboprobes not in annotated protein-coding genes are further examined for their protein-coding potential (see Methods for classification criteria). Excluding probes mapping to annotated coding exons and UTRs, we retain 141 intergenic and 10 intronic potential ncRNA transcripts that have predicted conserved local RNA structures (compared to 326 non-structured intergenic and intronic probes). Several RNA structures were found in known long ncRNAs such as *Xist, Miat, Meg3* and *Mirg*. Almost half (60) of the intergenic structured ncRNAs are more than 10 kb from the closest coding gene. Known RNA structures are annotated in nine putative ncRNA transcripts (4 microRNAs, 4 snoRNAs and *Xist*) and 80 structured UTRs (*e.g.*, 26 microRNAs, 19 snoRNAs, 6 SECIS, 6 Histone, and 3 IRES; see Additional file 2: Table S2). predicts 27 of these annotated RNA structures, whereas the other predictions are located up- or downstream from known structures.

### Spatial expression energy of structured transcripts

**Figure 2:**
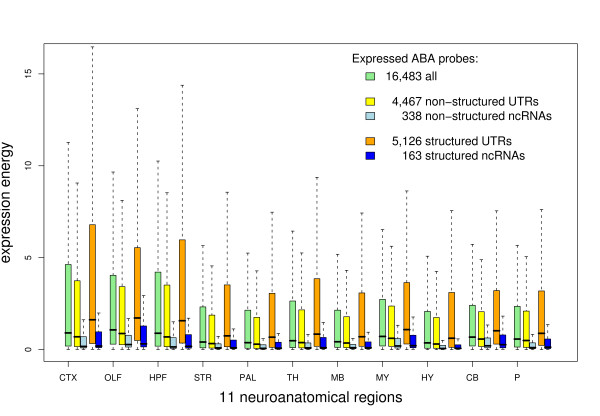
**Expression energy distribution of the Allen Mouse Brain Atlas probes.** Comparison of expression energy distribution of all expressed Allen Mouse Brain Atlas probes, structured and non-structured ncRNA and UTR probes in 11 neuroanatomical regions. Secondary RNA structures are predicted by . The box plot shows 1.5 interquartile range (dotted line), lower and upper quartile (box), and median (thick black line in the box). Brain region abbreviations: cortex, CTX; olfactory bulb, OLF; hippocampus, HPF; striatum, STR; pallium, PAL; thalamus, TH; midbrain, MB; medulla, MY; hypothalamus, HY; cerebellum, CB; pons, P.

The Allen Mouse Brain Atlas has mapped the ISH expression to defined regions (see Additional file 2: Figure S1). To identify neuroanatomical-specific patterns of structured transcripts and to possibly gain some insight into the biological function of these transcripts, we apply a multi-resolution hierarchical search of increasing levels of granularity that starts with 11 neuroanatomical regions (cortex, CTX; olfactory bulb, OLF; hippocampus, HPF; striatum, STR; pallium, PAL; thalamus, TH; midbrain, MB; medulla, MY; hypothalamus, HY; cerebellum, CB; and pons, P) in sagittal sections and ends with three-dimensional grids of voxels each 200 micron per side for both the sagittal and coronal plane [41]. Unless stated otherwise, by structured we refer to a *predicted* RNA structure. First, we compare the mean expression level (technically, “expression energy”, as defined in [9]; see Methods) of expressed structured probes versus expressed non-structured probes. The comparisons were performed separately in each of the 11 neuroanatomical regions, and for both the putative ncRNA and UTR categories. Expression of all expressed Atlas probes was examined as well. For this analysis, we used sagittal tissue sections because sagittal data is available for all probes whereas fewer (approximately 4,200 probes) have data in the coronal plane. In Figure 2, the major observations are that, first, structured UTR probes overall have the highest expression in the brain, and, second, structured ncRNA probes have higher expression than non-structured ncRNA probes, but are less strongly expressed than the average of all probes.

The significance of the observed patterns has been tested by different statistical methods (see Methods) with similar results. We addressed in which of the 11 neuroanatomical regions the mean expression energy of structured putative ncRNA and structured UTR probes is significantly different from expressed probes, intergenic and intronic, and UTR probes. The most striking result is that in all 11 neuroanatomical regions the 5,126 structured UTR probes have significantly higher expression than 4,467 non-structured UTR probes (see Additional file 2: Figure S2) as well as all expressed probes. The same applies for the finer level of granularity where the 11 neuroanatomical regions are further subdivided into 115 regions. On the other hand, there is significant expression enrichment of structured putative ncRNAs to non-structured ncRNAs only in cerebellum (using ).

Based on these observations we conducted further analyses to gain insight towards the possible causes of the enrichment of transcripts with structured UTRs. We studied significantly (*p*-value<0.001) enriched gene ontology (GO) terms [42] of UTR probes using functional annotation by DAVID [43]. We found support for function enrichment of binding (*p*=5E-40), localization (*p*=4E-18) and transport (*p*=4E-16) in structured UTR probes. Several GO terms for protein binding (*p*=1E-43) and RNA binding (*p*=7E-7) are significant for probes with structured UTRs (see Additional file 3), but none for non-structured UTRs (see Additional file 4). In addition, we found several GO terms which connect structured UTR probes to intracellular signaling pathways and suggest a directed RNA transport between nucleus and synapses or dendrites, *e.g.* the cellular components cytoplasm (*p*=2E-35), nucleus (*p*=6E-15) and synapse (*p*=7E-6) and the molecular functions intracellular signaling cascade (*p*=2E-18), protein transport (*p*=4E-11), protein localization (*p*=1E-11), vesicle-mediated transport (*p*=1E-11) and cytoskeletal protein binding (*p*=1E-6). For non-structured UTR probes there are four times less enriched GO terms, in general with lower significance than for structured UTR probes. Only the GO terms cytoplasm (*p*=3E-22) and transport (*p*=5E-4) are enriched for non-structured UTRs and are related to signaling function. Localization can imply different functional impact, for example direct involvement in transport, but it can also imply translational regulation at a specific subcellular location. Given the anatomy of the neurons where presumably many transcripts are located far away from the nucleus the observation of enriched expression of UTR regions with (predicted) RNA structures is consistent with this.

**Figure 3:**
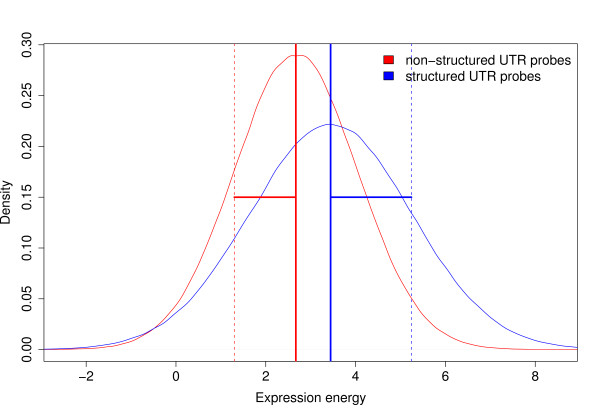
**Spatial expression divergence.** Mean normal distribution of expression energy of structured and non-structured UTR probes in 115 brain regions. The larger standard deviation (horizontal lines) of transcripts with structured UTRs shows their higher spatial expression divergence in the brain.

In embryonic cells it is known that the majority of localized RNAs are targeted to particular cytoplasmic regions by RNA elements and in mRNAs these are almost always in the 3’ UTR [44]. In brain cells our data agrees with these earlier observations in the way that 5’ UTR structures alone are not correlated to the binding, transporting and localization function of their protein products. We also mapped Atlas probes overlapping UTRs to a list of 76 active proteins in synapses [45]. A significantly greater number of their mRNAs has a structured UTR (Fisher’s exact test *p*-value = 0.0023; see Additional file 2) which supports a role for the UTR structures as functional RNA elements. Another supporting observation for localized RNAs with structured RNA elements is their higher spatial divergence in the brain described by larger deviation of expression in 115 neuroanatomical regions (see Figure 3). Alternatively, spliced UTR transcripts may act independently from their host mRNA. However, this case cannot be verified without further examination of the probe captured transcripts.

The expression data also shows slightly higher expression of structured putative ncRNA transcripts than non-structured ncRNAs in many brain domains as indicated in Figure 2 and Additional file 2: Figure S3. Mean expression of the 151 structured ncRNA candidates is larger in 83 out of 115 brain regions. Enrichment is significant in 15 regions (including cerebellum) compared to 3 regions with significantly enriched non-structured ncRNA probes using a more robust measure of location assuming dependency between multiple ISH measurements (; see Methods).

It is essential to determine whether the presence of enriched transcripts is due to slower degradation caused by RNA structures [46]. The delay of degradation of transcripts folding in conserved RNA structures may support an increased half life of brain relevant RNAs. Proteins are actively synthesized in neuronal synapses despite the long distances between nucleus and synapses. For this purpose translational control of gene activity appears to be more efficient than transcriptional control [47]. Conservation of different structures in different transcripts suggests that they are involved in a rich variety of post-transcriptional regulatory interactions, *e.g.* through altered transcriptional stability. Combined with the previously described GO analyses, this suggest that proteins involved in molecule mobility are produced in larger numbers, and mRNAs and ncRNAs are transported to their intended cell destination before carrying out their function.

### Protein binding of structured UTRs

As an initial step towards assigning functional information we searched for proteins that may bind to predicted structures in UTR regions. RNA binding proteins are trans-acting factors that function, *e.g.*, in RNA localization. For instance, the mRNA of the neurotrophic tyrosine kinase *TrkB* receptor is transported to dendrites and translated in response to neural activity. The mouse *TrkB* 5’ UTR contains one conserved and one mouse-specific single internal ribosomal entry site (IRES) whose RNA secondary structures and sequence-specific motifs are proposed to be integral to IRES-dependent translation [48]. In agreement with this, the prediction finds the conserved IRES structure in 9 mammals, whereas, as expected, the unconserved IRES structure was not predicted. The structure consists of two stems of which the 3’ stem is the same as previously shown in human [48]. Activity of the conserved IRES is enhanced in the presence of the polypyrimidine tract binding protein *PTB1*[49]. In the ISH data correlated expression of *TrkB* and *PTB1* can be seen, even though at a low level, in the olfactory bulb (*ρ*=0.49) and medulla (*ρ*=0.52) using the spatial homology search tool [41] (see Methods).

In comparison to non-structured UTRs, a correlation-based search for similar expression pairs (using ) results in slightly more correlated expressed pairs between transcripts coding for RNA binding proteins and transcripts with structured UTRs. To identify spatial and brain-wide correlations, we used Pearson’s correlation coefficient greater than a threshold of _*ρT*_=0.9 and _*ρT*_=0.85, respectively (see Methods for the selection criteria of _*ρT*_’s and spatial expression). We identified spatial correlation between 41 RNA binding proteins annotated in RBPDB [50] and 66 structured UTR transcripts mostly in thalamus, pallium and hippocampus (see Additional file 2: Table S3), as well as brain-wide correlated expression between 6 RNA binding proteins and 12 structured UTR probes (see Additional file 2: Table S4). We also searched for potential interaction sites of RNA binding proteins around UTR structures which are discussed below.

### Correlated expression between structured transcripts

By examining correlated expression patterns, we can hypothesize new functions for previously uncharacterized structured transcripts or identify potential interacting RNA molecules as well as RNA-protein interactions due to localized translation as described above. The following prediction of an annotated UTR element exemplifies connectivity of functional related molecules. We predict a widely conserved (in 16 organisms from human to zebrafish) 25 nt stem-loop in the 3’ UTR of rat brain-derived neurotrophin factor *BDNF*. This stem-loop partly overlaps the loop and 5’ end of the annotated core region of an extended stem-loop previously predicted in the full-length UTR structure (by ) [51]. The 3’ UTR structure of *BDNF* provides a scaffold for interaction of various RNA binding proteins, polyadenylation factors and miRNAs in response to *C*^*a*2 + ^ signal (neuron activity). The interaction results in *C*^*a*2 + ^signal-dependent stabilization of mRNAs in neurons [51].

**Figure 4:**
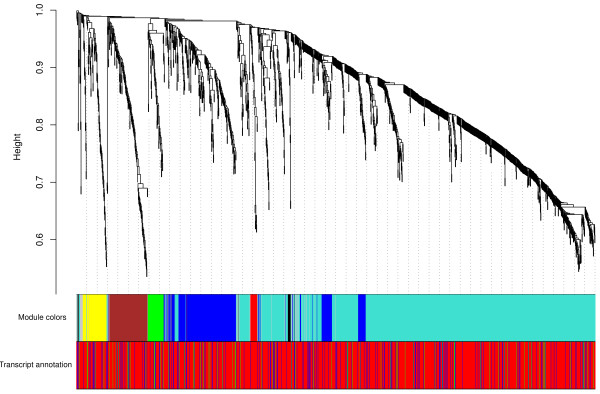
**Expression profile clusters of structured UTR probes.** Hierarchical clustering of coronal expression energy profiles in 115 neuroanatomical regions of quality selected images of 165 Allen Mouse Brain Atlas probes with predicted 5’ UTR structures, 1,188 probes with predicted 3’ UTR structures and 66 probes with predicted structures in both UTRs. Probes within an individual module have similar expression patterns. The brain area(s) with the strongest correlated expression pattern(s) for each of the 8 modules are: isocortex (turquoise module), dorsal thalamus (yellow), epithalamus (gray), motor-related pons and midbrain in the brain stem (blue), striatum (brown), cerebellum (red), and midbrain (green and black). Probes in each module have additional (weaker) correlated expression pattern(s) in other brain areas and the turquoise, blue and black module represents probes with correlated expression patterns in the entire brain. The color coding of genomic locations (Transcript annotation) shows transcripts with a 5’ UTR structure as blue bars, transcript with a 3’ UTR structure as red bars and transcripts with predicted structures in both UTRs as green bars.

**Figure 5:**
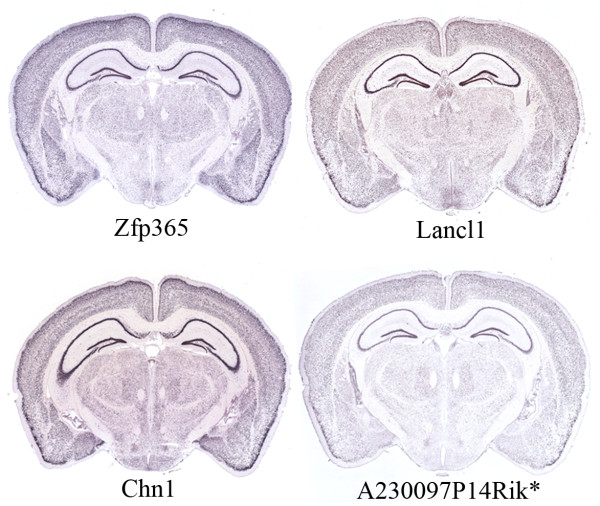
**In situ hybridization of an RNA binding protein and correlated expressed probes.** A representative coronal section showing in situ hybridization data from *Zfp365*, *Lancl1*, *Chn1*, and A230097P14Rik*. All probes show strong widespread expression throughout the brain.

Before studying gene pairs of correlated expression we look for groups of transcripts with structured UTRs with similar expression patterns in 115 brain regions. High quality probes with coronal data (165 probes with structures in 5’ UTRs, 1,188 probes in 3’ UTRs and 66 probes in both UTRs; see Methods for selection criteria) are clustered in modules of correlated expression [52]. Most structured UTR probes have correlated expression patterns over the entire brain with the strongest signals in the isocortex (turquoise bar in Figure 4) and motor-related areas in the brain stem (blue bar). The strongest spatial pattern occurs in epithalamus (grey), followed by cerebellum (red), striatum (brown) and midbrain (green).

 is used to study correlated expression of structured Atlas probes in the entire brain. Starting with high quality probes we found 78 structured UTR transcripts with strong brain-wide expression involved in 352 brain-wide correlation pairs (threshold _*ρT*_=0.85; see Additional file 2: Figure S4 for correlation network). Strong spatial activity is obtained for 264 structured UTR probes involved in 1,898 local correlation pairs (_*ρT*_=0.9). Many transcripts have correlated expression to only a small number of other transcripts (see Additional file 2: Figure S5). One such example is the *Zfp365* zinc finger protein which is brain-wide correlated expressed to *6530418L21Rik* (*ρ*=0.86), the signal transduction protein *Chn1* (*ρ*=0.87) and A230097P14Rik* (*ρ*=0.86), whose mRNAs have highly conserved 3’ UTR structures. Representative ISH images of some correlated probes are shown in Figure 5.

**Figure 6:**
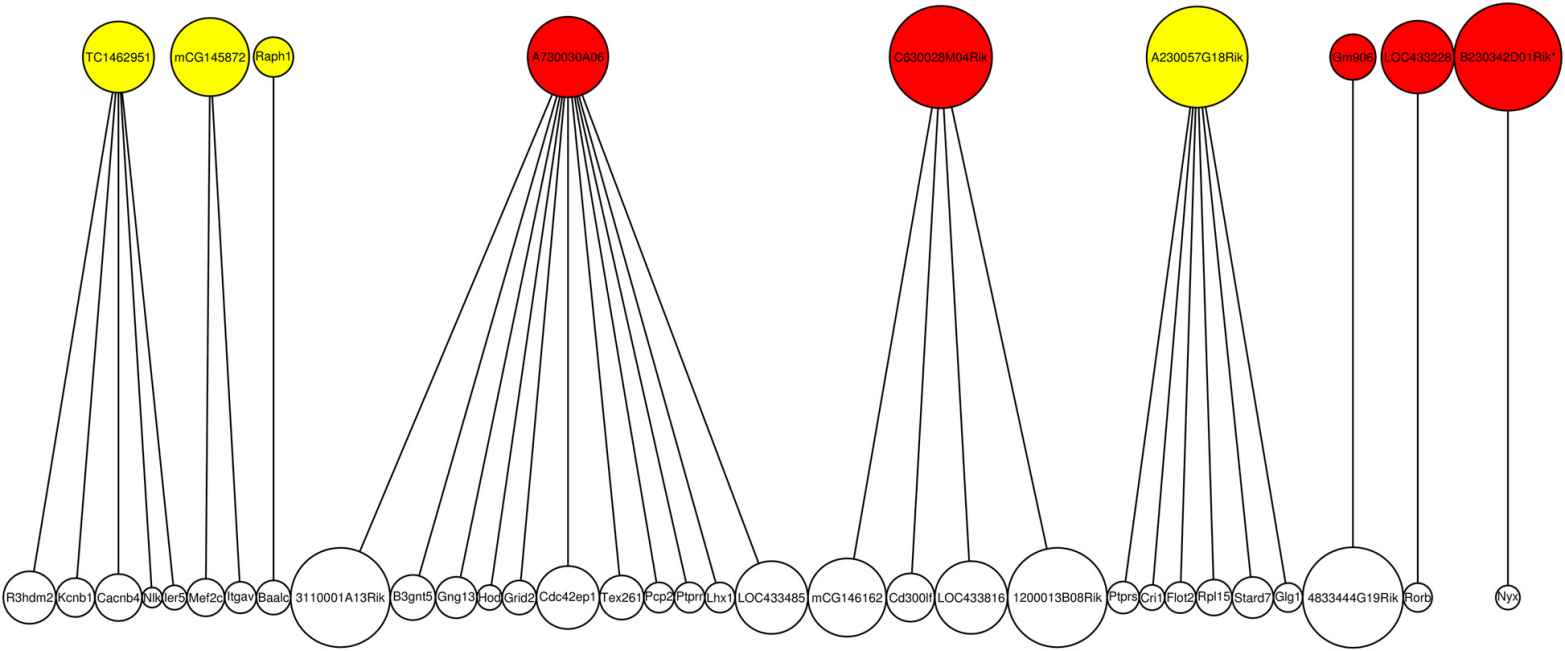
**Network of brain-wide correlated expression patterns containing ncRNAs.** Correlation network of 9 putative ncRNA transcripts with correlated expression over the entire brain. Red nodes represent transcripts without RNA secondary structure predictions and yellow nodes with structure predictions. These 9 transcripts are involved in 33 correlation pairs (edges, *ρ*>0.8).

Sagittal image data was included for correlation pair analysis of ncRNAs. Of 477 putative ncRNAs, 9 show strong brain-wide correlated expression in 33 correlation pairs (_*ρT*_=0.8) including 4 structured ncRNA candidates (*mCG145872*, *A230057G18Rik*, *TC1462951* and *Raph1*; see Additional file 2: Table S6 for a list of all correlated pairs). Most of these transcripts are involved in small cliques of correlated expression, see Figure 6. Additional file 2: Figure S6 shows representative ISH images of the non-coding myocardial infarction associated transcript *A230057G18Rik* (*Miat*) and its correlated expressed transcripts. More often ncRNA correlated expression appears in restricted brain domains rather than brain-wide. Mostly small cliques of correlated expressed transcripts are found for 134 ncRNAs (326 correlation pairs) including 33 structured ncRNA candidates involved in 84 correlation pairs (_*ρT*_=0.9; see Additional file 2: Table S7 and Figure S7). Spatial correlation patterns also exist for known microRNAs and snoRNAs targeted by intergenic riboprobes. For instance, *mir-101a*, which is encompassed by AK021368 (*E130102H24Rik*), has correlated expression in hindbrain (*Emg1*) and pons, *mir-154* and *mir-410* which are encompassed by *Mirg* are expressed brain-wide with regional covariance in pons (*Mtch1*) and hippocampus (*Mrpl13*), and the *ACA17* snoRNA hosting transcript *mCG1030139* is correlated to *Mtnr1b* in the thalamus.

### Thermodynamic stable RNA-RNA interactions

Many known ncRNAs exhibit their functionality through binding of RNA target sequences, such as microRNAs bind mRNAs, snoRNAs bind ribosomal and small nuclear RNAs, and certain lncRNAs may bind microRNAs [53] to regulate their activity or guide RNA editing. Potential RNA-RNA interactions between structured transcripts and correlated expressed RNAs were searched by scanning all putative ncRNAs and UTRs of Atlas transcripts for statistically significant intermolecular RNA binding sites. By combining [54] and [55] we calculate the minimum free energy (MFE) of putative interaction sites in the real data, and the same strategy was used to create background distributions on dinucleotide shuffled data for *p*-value calculation (see Methods).

For 6 putative ncRNAs with local and 2 putative ncRNAs with brain-wide correlated expression we found putative interaction sites to 3’ or 5’ UTR of the correlated mRNAs, however, of relatively large *p*-values (see Additional file 2: Table S8). For instance, a non-conserved interaction site is predicted between the putative ncRNA *TC1462951* and the 3’ UTR of *Kcnb1* (see Additional file 2: Figure S8 for ISH image and expression mask). The putative ncRNA *LOC433503* may interact with a conserved region in the 3’ UTR of *Gpx3*, only 100 nt upstream of the common stem-loop structure *SECIS* (see Additional file 2: Figure S9). In addition, around 600 significant (*p*-value<1e-05) interaction sites with a MFE smaller than -40 kcal/mol are predicted by and between structured putative ncRNAs and, *e.g.*, UTRs of mRNAs coding for RNA binding proteins (*Rbpms* and *Samd14*; see Additional file 5), but the ISH data does not reveal correlated expression.

## Discussion and Conclusions

Microarray studies have shown that at least 50% of assayed transcripts are expressed in the brain [56], with up to 80% of transcripts shown to be expressed by ISH [9]. In order to gain a better understanding of transcripts in the brain that may be contributing to brain function, we examined which transcripts have an RNA structure. We observed that in silico predicted RNA structures are enriched both in coding (UTR regions) as well as noncoding transcripts in almost all regions of the adult mouse brain. The simplest interpretation of the data is that the Atlas probes showing higher expression are enriched for predicted RNA structures. Through the integration of mouse brain expression data and secondary RNA structure predictions, we found that transcripts with such predictions in their UTRs, those that are enriched in the 3’ UTR adjacent to the ORF, have the highest expression throughout the brain. Many of these mRNAs as well as their protein products may act as signaling molecules whereas the UTR structures serve as binding motifs for other RNAs and proteins involved in intracellular signaling pathways. This hypothesis is supported by (i) enriched gene ontology terms binding, transport and localization, (ii) correlated expression patterns between mRNAs with structured UTRs and RNA binding proteins, and (iii) a larger expression diversity of transcripts with structured UTRs. UTR structures as signal for motor-driven transport and translational repression through RNA binding proteins are especially attractive in neurons where the transport of information stored in ribonucleic sequences from the nucleus through long axons to the synapses is an important component of neuronal functionality [47].

We investigated this hypothesis further by searching for potential protein binding motifs around (predicted) UTR structures to 72 RNA binding proteins annotated in RBPDB [50] (see Methods and Additional file 2). The majority (90%) of the UTR structures has at least one predicted binding motif in its neighborhood (see Additional file 2: Table S5). These motifs can be bound by 21 proteins. Only 9 proteins, however, have significantly more predicted targets than expected by chance, and half of the binding proteins are involved in splice site regulation. The analysis indicates that some interesting binding motifs can be found, such as neural-specific *Elavl2*, cytokine’s degrading *Zfp36*, and mRNA trafficking *Khsrp*. *Zfp36* binds AU-rich elements (ARE) in the 3’ UTR of some cytokine mRNAs and promotes their degradation. Intriguingly, an AU-rich region (AU content of 85% over a length of 41 nt) starts at the 3’ end of the predicted UTR structure of *6530418L21Rik* (see Additional file 2: Figure S10) and its expression is highly correlated with that of another zinc finger protein (*Zfp365*) and *Lancl1*, an RNA binding protein involved in immune surveillance of the brain [57] (see Figure 5). Assuming that *6530418L21Rik* works as a signaling molecule, its transport function may be deactivated through the binding of *Zfp36* close to its 3’ UTR structure. However, here a large scale investigation in RNA-protein binding is still limited due to the low information content of binding motifs described by short sequence-based position weight matrices (PWMs).

Motivated by the GO analysis we also considered the hypothesis that structured RNAs in neural cells are themselves involved in establishing intracellular signaling pathways. For instance, Dienstbier *et al.*[3] provide evidence that Egalitarian (*EGL*) and the dynein cofactor Bicaudal D (*BICD*), previously known to be required for minus-end-directed mRNA transport, mediate linkage of various mRNAs to the dynein motor in Drosophila melanogaster. Here, we show that *EGL nine homolog 1* and *BICD* have predicted UTR structures, BICD is associated with the GO terms intramolecular, cytoplasm, localization, transport and binding and EGL with the GO term binding. Proteins, such as EGL, BICD and cytoskeletal protein filaments, are needed to establish intracellular pathways for directed cytoplasmic RNA transport towards synapses and dendrites. For signal propagation in the opposite direction back to the nucleus, mRNAs coding for these proteins have to be transported first and, thus, need cis-acting RNA elements too. The hypothesized directed RNA transport is illustrated in Additional file 2: Figure S14.

We also looked for predicted RNA structures in all UCSC and RefSeq annotated UTRs of protein coding genes overlapped by Atlas probes. We found 9,378 of these genes with RNA structure predictions in their UTRs and 5,576 without UTR structures. Of the 4,467 Atlas probes that overlap unstructured UTRs, 1,246 probes have a structure elsewhere in (at least one variant of) the UTR. It is unknown whether these structures are present in brain. Assuming they are, i.e., reclassifying as “structured” some of the UTR probes previously classified as “unstructured”, we see even larger differences between the expression of structured and non-structured UTR probes (see Additional file 2: Figure S13 compared to Figure 2). Hence, we conclude that our overall statistics also hold for RNA structure annotation in full-length transcripts. In addition, we showed that putative ncRNAs with locally predicted RNA structures have significantly higher expression than non-structured intergenic and intronic transcripts in several brain regions. Positive correlated expression patterns between pairs of transcripts are often domain-specific for putative structured ncRNAs. Most promising are 4 ncRNAs with brain-wide correlated expression in small cliques (*mCG145872A230057G18RikTC1462951*, and *Raph1*; see Additional file 2: Table S6), and several ncRNAs with only one spatially correlated expressed transcript. We investigated conditions where RNA structure has a function, such as RNA-RNA interactions between correlated expressed RNA transcripts. One of the applied methods in this study, *e.g.*, predicts the interaction site of two sequences. However, it is known from RNA motif searches that short sequence motifs can often appear by chance which partly explains the large *p*-values for the predicted RNA-RNA interactions. Consideration of homologous sequences in other species and duplex folding by using tools such as [58,59] may help to obtain more significant predictions.

A major uncertainty is the limited resolution of the informatics detection of expression in the ISH images and, thus, the correlation data. Several cells comprise a single voxel leading to interpolation between expression information and noisy expression energy. Sagittal images are more impacted by registration errors since only a single hemisphere is available for registration. The majority of correlation pairs detected in the sagittal plane failed validation by manual inspection of the ISH images (see Methods for further information). The largest cliques of correlated expression are often because of process artifacts in the images or the absence of expression (see Additional file 2: Figure S7). One desirable quality improvement of the correlation data is the weighted consideration of the voxel neighborhood which would improve the confidence in correlated expressed pairs by sacrificing some level of detail. Furthermore, the data might also be interesting for graph theoretical analyses on gene expression correlation networks. Features of these networks are relatively unknown and the correlation coefficient threshold could be more sophistically chosen by analyzing its influence on network connectivity. The large number of 3’ UTR probes might also target ncRNAs, in addition to the untranslated region of mRNAs. In several specific cases we observed highly correlated brain-wide expression, *e.g.*, between the 3’ UTR probe *Kcnc2* and its intronic *mCG142089*, and between *Dusp3* and its downstream-sense located structured probe *TC1462951*, but these probe pairs may have bound the same (pre-spliced) transcript. Thus, conclusions about correlated expression of adjacent or overlapping transcripts are hardly possible, especially if they have widespread expression throughout the brain.

An additional concern is that the observed correlation between structure and expression level might be an artifact of RNA degradation. All exonucleases have problems initiating degradation close to stable stem structures [60]. Hence, the abundant enrichment of transcripts hosting RNA structures may be at least partly explained by their slower degradation and, thus, higher accessibility to riboprobes compared to transcripts lacking RNA structure. In fact, if the structures are involved in translational regulation, reduced degradation is just as effective as increased transcription in terms of raising steady-state transcript levels. Thus, to determine when *e.g.*, a bound protein primarily serves to regulate or primarily serves to prevent degradation seems hard, in particular if preventing degradation is part of the regulatory mechanisms as is the case with the iron metabolism in vertebrates [61]. However, the observed enrichment of transcripts with structured UTRs is not related to a particular structure, hence, it is unlikely that a particular RNA binding protein that promotes transcript stability by binding to a specific structured RNA motif is responsible for the broad expression pattern.

A final concern is that our results might be explained by a difference in the hybridization efficiency of Atlas probes towards structured versus unstructured transcripts. Hybridization is affected by a variety of factors, such as probe accessibility and affinity to the targeted molecule. For short oligos, although there are some contexts in which hybridization may be enhanced by appropriate RNA structures [62], it is most often suggested that highly structured regions in a target transcript would reduce hybridization efficiency. Many riboswitches, for example, down-regulate translation by sequestering the ribosome binding site in a structure that blocks interaction with the 16S rRNA [63]. This evidence suggests that structured target molecules would generate a decreased signal, but we observed an increase. In addition, Atlas probes were chosen to be 400-1200 bases in length. For such long probes that are perfectly complementary to their targets, the fully hybridized “double helix” will be the most energetically favorable state and seems likely to form easily from a simple initial toe-hold/zipper extension interaction from almost any initial conformation of the target. Thus, on balance, it does not seem likely that riboprobe affinity to structured versus unstructured transcripts explains the observed enrichment of structured transcripts.

Overall, our results show a huge potential for RNA structure as an abundant and active feature on both coding and noncoding transcripts in the adult mouse brain. Using we predicted more than 40,000 RNA structures (mostly in intronic and 3’-untranslated regions) in about 10,500 expressed Atlas probes in the adult mouse brain. Even though *in silico* methods for RNA structure prediction hold high false positive rates of up to 50% [33,34] our findings still leave room for functional RNA structures in the Atlas transcriptome data. The significantly enriched expression energy of structured transcripts is hard to explain by chance and supports the theme of functional RNA structures in the mouse brain. In the future, a structure analysis remains to be carried out on a global transcriptome data set in the adult mouse brain because the Atlas data primarily focus on protein-coding transcripts and has limited data on noncoding transcripts.

## Methods

### 

#### Mapping and classification criteria

The Allen Mouse Brain Atlas (Atlas) probes have been previously mapped to the mouse (mm8) genome [36]. Probe coordinates and RNA structure predictions are mapped to UCSC [39] and RefSeq [40] gene tracks with at least 10% overlap of probes and predictions. Intergenic and intronic probes are further checked for significant protein-coding potential as performed by Mercer *et al.*[36]: CRITICA [64] predicts significant protein-coding potential in the probe sequence or any targeted transcript, and ORFs greater than 120 codons are detected that comprise at least one third of the transcript length. In addition, we applied [65] on mm8 based UCSC multiz17way alignments of intergenic and intronic probes to also detect shorter conserved ORFs (*p*-value<0.001).

RNA structure predictions are in general unclear about which strand actually contains the structure [34]. Therefore, strand predictions of RNA structures were not used. We assume that a prediction on one strand yields a candidate on both strands. We mapped structures to Atlas probes if the structure overlaps at least 1nt of an intergenic probe or if the structure overlaps at least 1nt of a UTR exon, coding exon, or intron that was mapped to the Atlas probe. We used this rather conservative procedure instead of mapping to putative respective transcripts of the probes to avoid counting splicing variants with predicted RNA structures. This procedure will miss some structured UTRs, however, our statistical conclusions still hold for the investigated subset of UTR structures.

### 

#### Known RNA structures

The Allen Mouse Brain Atlas probes are annotated as known structured RNAs if they overlap at least 10% of a mouse microRNA in miRBase v10.0 [66] or a human track in miRBase, snoRNABase [67], Rfam 9.1 [68], ncRNA.org or Jones’ and Eddy’s ncRNA list [69]. We used generated alignments and chained blastz alignments (liftOver tool) to map the human tracks to its mouse homologs.

### 

#### Allen Mouse Brain Atlas technical information

The expression energy quantifies the overall expression at a given voxel. It is calculated as the product of expression level and density of cells expressed in that voxel [41]. All riboprobes have sagittal expression data and a subset of riboprobes have both sagittal and coronal expression data. Informatics processing of the expression data from the sagittal sectioning plane is, however, effected by the data only containing one hemisphere (coronal data has two hemispheres), various starting and ending positions of the tissue sections processed for an individual riboprobe, and minor variability in the section cutting angle. In contrast, coronal data typically registers better as the symmetry of the section helps to lock the other two dimensions of the 3D grid together. To increase the accuracy of expression profiles and to meet quality control metrics, we created a high quality dataset that includes 1,525 structured UTR probes from Table 1 with coronal image series minus 125 coronal images series having manual detected processing artifacts (such as upside down images), widespread expression or missing image data due to failure of individual tissue sections.

### 

#### Robust statistics

Significant spatial expression patterns are found by two sample location t-tests of the null hypothesis that the expression energy means of two sets of Atlas probes are equal. Errors associated with each ISH measurement are not totally independent from each other, thus, the normal distribution assumption does not hold. We apply bootstrap procedures to estimate the unknown distribution of expression energy in neuroanatomical regions. The percentile-t bootstrap *p*-values differ from ordinary percentile *p*-values in that they are based on bootstrap approximations of the distribution of the studentized estimator rather than the distribution of the original estimator. P-values are adjusted by the method of Benjamini & Hochberg to control the false discovery rate and the null hypothesis was rejected if the adjusted *p*-value < 0.25. As a more robust measurement of location we also calculated adjusted *p*-values of 0.2*%* trimmed means using the bootstrap methods and [70].

### 

#### NeuroBlast and Pearson’s correlation coefficient

The Atlas provides interpolated expression energy in regular 3-dimensional lattices of cellular resolution for each sagittal and coronal image series. The correlation of the expression energy for each probe pair is calculated by the spatial homology search tool [41]. calculates the Pearson’s correlation coefficient *ρ* between two vectors of two probes that hold the expression energies for all voxels each 200 micron per side in a defined brain region. The cumulative frequency distribution of the number of correlation pairs over *ρ* follows typically a negative sigmoid curve (see Additional file 2: Figure S11), thus we chose a threshold _*ρT*_close to the right flattened area of the curve for selecting the most promising correlation pairs. Spatial correlations have tendential higher *ρ*’s than brain-wide correlations due to the lower amount of compared voxels. Hence, we chose _*ρT*_ slightly higher to select spatial correlations.

### 

#### Spatial expression

Riboprobes with high spatial expression are defined as probes with larger relative expression in one brain domain compared to the entire brain:

(1) $$\frac{v_{d}(E\geq 2)}{v_{d}}\;/\;\frac{v_{b}(E\geq 2)}{v_{b}}>1,$$

where _vd_ is the number of voxels in one domain and _vb_is the number of voxels in the entire brain.

### 

#### Protein binding sites

UTR structures and their 50 nt flanking regions are searched for potential protein binding motifs using RBPDB [50]. First, position weight matrices (PWMs) from RBPDB were used together with the perl TFBS library [71] to scan sequences for binding sites to 72 RNA binding proteins with expressed Atlas probes (461 proteins in RBPDB). Second, we sequence aligned (BLAT) our sequences against 1,021 individual RNA sequences from single-sequence experiments excluding consensus (IUPAC) sequences.

### 

#### Prediction of significant RNA-RNA interactions

Potential interaction sites of all ncRNAs included in the Atlas are searched in all UTRs of Atlas transcripts annotated in mouse by UCSC or RefSeq. Probabilities of local basepairs are calculated by in all sequences. These probabilities are taken as input for for considering sequence accessibility. v0.2 is used with the parameters . We calculate a *p*-score for putative interaction sites which is the probability of obtaining a MFE *S* by chance greater than the observed MFE. Therefore, we dinucleotide shuffled 100 times all queries and targets of the top 10,000 interaction pairs and calculated their binding MFE. Additional file 2: Figure S12 shows that the MFEs are extreme-value distributed (evd) with a maximum around -10 (which is used as censored cutoff for evd parameter estimation). Since MFE is highly dependent on the length and GC content of the interaction site, we describe the background distribution (*λ* and *μ*) for 49 combinations of the two covariates using the package [72] and estimate a *p*-value of predicted RNA-RNA interactions on the appropriate background by the equation:

(2) $$P(S\geq \mathrm{MFE})=1-\exp(-e^{\lambda (\mathrm{MFE}-\mu )})$$

## Competing interests

The authors declare that they have no competing interests.

## Authors’ contributions

SES did the analyses and wrote the main part of the manuscript. SMS and MJH helped in the integration of the Allen Mouse Brain Atlas expression data, and SMS manually inspected the ISH images. JG and WLR had the idea for the study and helped to integrate the RNA structure predictions. All authors contributed to the manuscript.

## Supplementary Material

Addtional file 1 **Annotation of predicted structured probes.** CSV-file listing the features of all structured probes from Table 1 and their CMfinder predicted RNA secondary structures. Click here for file

Addtional file 2 **Tables and figures.** This file contains lists of correlated expressed structured riboprobes, and additional tables and figures. Click here for file

Addtional file 3 **GO analysis of structured UTR probes.** 4115 structured UTR probes with known gene symbols are examined for GO term enrichment. Click here for file

Addtional file 4 **GO analysis of non-structured UTR probes.** 3407 non-structured UTR probes with known gene symbols are examined for GO term enrichment. Click here for file

Addtional file 5 **Predicted significant RNA-RNA interactions.** CSV-file listing 585 significant (*p*-value<1e-05) interactions between structured putative ncRNAs and UTRs. The interaction sites are predicted by RNAplfold and RNAplex to be larger than 9 nt and with a MFE smaller than -40 kcal/mol. Click here for file
