# Supplementary material for: Transcripts with in silico predicted RNA structure are enriched everywhere in the mouse brain
Source: BMC Genomics. 2012 May 31;13:214. doi: 10.1186/1471-2164-13-214 (PMC3464589; doi:10.1186/1471-2164-13-214)
Supplement: Addtional file 2 — Tables and figures. This file contains lists of correlated expressed structured riboprobes, and additional tables and figures. [file 1471-2164-13-214-S2.pdf]

Additional file 1

Transcripts with *in silico* predicted RNA structure are enriched everywhere in the mouse brain

Stefan E Seemann, Susan M Sunkin, Michael J Hawrylycz, Walter L Ruzzo, Jan Gorodkin

Additional Information

P-value estimation

The *P*-values are estimated in a similar way as in Torarinsson *et al.* (2008). An observation is significant if the observed number of hits cannot be described by chance. The *P*-value is calculated using the normal approximation to the binomial distribution that is implemented in `R` by the function `pnorm`

$$P[X > x] = \text{pnorm}(\text{M}, \mu, \sigma, \text{lower.tail=F})$$

where M is the observed number of non-overlapping RNA motifs that overlap the target set, $\mu = N \times Pr$, $\sigma = \sqrt{N \times Pr \times (1 - Pr)}$, Pr is the probability of each RNA motif to hit a target, and N is the number of all RNA motifs.

Fisher's exact test for enrichment of structured UTR transcripts in synapses

We found 27 genes with structured UTRs (out of 5,122 structured UTR probes) and 10 genes without UTR structures (out of 4459 non-structured UTR probes) that are known to be located in synapses (out of 76 proteins known to be located in synapses). The significance of more proteins in synapses coded by mRNAs with structured UTRs is tested by Fisher's exact test using the `R` function `fisher.test(data,alternative="g")`: *p*-value = 0.002285

	Structured UTR	Non-structured UTR
Synapse	27	11 = round(10*(5122/4459))
Non-synapse	49 = 76-27	65 = round(76-10*5122/4459)

Structured UTRs and RNA binding proteins

21,269 CMfinder predicted structures are located in UTRs (strand unspecific) and have an average length of 75 nts. These UTR structures and their 50 nts flanking regions are searched for potential protein binding motifs collected in RBPDB (Cook *et al.*, 2010). First, we used the perl TFBS library (Lenhard and Wasserman, 2002) to scan our sequences against position weight matrices (PWMs) from 72 RNA binding proteins, and second, we sequence aligned (BLAT) our sequences against 1,021 individual RNA sequences from single-sequence experiments excluding consensus (IUPAC) sequences. The protein binding sites are correlated with their position in CMfinder motifs, whereas protein binding sites in stems are defined to cover more or equal than 50% base pairs. In approximately 90% of UTR structures, at least one binding motif was found to only 21 proteins. Using DAVID we found "RNA metabolic process" (p-value = 2.5E-17) as the second most significant gene ontology term directly after "RNA binding" (p-value = 1.1E-18). The binding sites are enriched neither in the 5' flanking region, the RNA motif, nor the 3' flanking region. The RNA binding protein *Vts1* mostly binds UTRs in stem regions, whereas the binding motifs of the other proteins are mostly found 5' and 3' of the predicted UTR structures (see Supplementary Table S4). Surprisingly, motifs inside UTR structures appear more frequently in stems than in loop regions.

Additional Tables

Table S1: Probabilities (P-values) that the overlap of `CMfinder` candidate regions and Allen Mouse Brain Atlas (Atlas) probes can be explained by chance. The rows stand for (1) the considered lower (exclusive) and upper (inclusive) boundary of considered *GC contents*; (2) the number N of all RNA motifs; (3) the amount of *Atlas probes*; (4) the probability Pr of each RNA motif to hit a target; (5) the mean value $\mu = N \times Pr$; (6) the standard deviation $\sigma = \sqrt{N \times Pr \times (1 - Pr)}$; (7) the observe number M of non-overlapping RNA motifs that overlap the target set; and (8) the calculated P-value using the normal approximation to the binomial distribution (Torarinsson *et al.*, 2008).

gc content	total	(0-35]	(35-40]	(40-45]	(45-50]	(50-100]
N	434,526	77,107	51,677	58,382	71,147	175,354
Atlas probe	23,016	493	1,522	3,719	6,134	11,148
Pr	0.014690	0.001206	0.004832	0.011618	0.021423	0.040831
μ	6,383.257	92.966	249.686	678.278	1,524.188	7,159.837
σ	79.306	9.636	15.763	25.892	38.620	82.870
M	13,362	596	756	1,089	1,852	9,101
P-value	0	0	1.16×10^{-226}	5.73×10^{-57}	1.05×10^{-17}	1.21×10^{-121}

Table S2: Known functional RNA motifs in Allen Mouse Brain Atlas probes with `CMfinder` predicted secondary RNA structure. `CMfinder` motifs that overlap the annotated RNA motif are listed in the third column.

Expressed structured putative ncRNAs with known RNA motif		
Atlas probe	Known RNA motif	`CMfinder` motif
Mirg	mmu-mir-410;mir-154	
LOC381754	mir-544	
mCG1030139	ACA17	
mCG1044749.1	SNORD49;SNORD65	
0610007N19Rik	SNORD123	
9430008C03Rik	ACA39;ACA60;SNORA71	
4933416E14Rik	mir-692	
E130102H24Rik	mmu-mir-101a	
Xist	Xist	

Expressed probes with UTR structures with known RNA motif		
Atlas probe	Known RNA motif	`CMfinder` motif
Hist1h2ac	Histone3	
A930034L06Rik	ACA39;ACA60;SNORA71	
E130103I17Rik	mir-220	
Samd12	ACA17-related	
9630011N22Rik	7SK_RNA-related	
Egfl7	mmu-mir-126	
Asgr1	hsa-mir-195	
Atp6v0e	U19-2	
Bag1	IRES_Bag1	mm8chr4/41136750-41136844
Bet1	SNORA40	
Csf3	G-CSF_SLDE	mm8chr11/98519181-98519449
Eif4a1	HBI-61;snoU6-77;U67;SNORA63;SNORA4	
Eif4a2	SNORA63;SNORA4;SNORA81	
Fgf13	mmu-mir-504	
Ctgf	CAESAR	
Fmr1	SNORA25	
Gpx2	SECIS	mm8chr12/77711209-77711380
Gpx3	SECIS;Histone3	mm8chr11/54753593-54753702
H3f3a	Histone3	
0610005G16Rik*	Mitoch._tRNA-derived_pseudogene; SRP_RNA-related	mm8chr11/54753593-54753702
0610006F12Rik*	Mitoch._tRNA-derived_pseudogene	mm8chr1/24567364-24567508
Hebp1	ACA17-related	
D830047O19Rik*	Mitoch._tRNA-derived_pseudogene	mm8chrM/11435-11752
E330014M11Rik*	Mitoch._tRNA-derived_pseudogene	mm8chrM/11435-11752
Igf2	mmu-mir-483;IRES_IGF2	
Kcnc1	K_chan_RES	
Kcns2	K_chan_RES	
Krt36	mir-492	mm8chr11/99920569-99920618
Lhx2	mir-1302	
mt-Nd3	Mitoch._tRNA-derived_pseudogene; SRP_RNA-related	mm8chrM/7533-7853
Npn1	mir-199a-2;mir-214	mm8chr1/164060043-164060137; mm8chr1/164054462-164054592
Oaz1	Antizyme_FSE	mm8chr10/80231399-80231516
Prnp	Prion_pknot	
Psmb5	mmu-mir-686	
Ranbp1	SNORA77	
Sepp1	SECIS	mm8chr15/3229965-3230081

Table S2: continued

Expressed probes with UTR structures with known RNA motif		
Atlas probe	Known RNA motif	`CMfinder` motif
Sepw1	SECIS	
Synj2bp	SNORA40	
Map2k4	hsa-mir-744	
Zfp275	U13-related	
Sepx1	SECIS	
Hist1h1e	Histone3	mm8chr13/23629246-23629295; mm8chr13/23628150-23628191
Fbxo8	mir-692	
Ywhab	ACA51-related	
Phca	U87	
Cks2	ACA2b;ACA34	
1810032O08Rik	snR38A;R38c	mm8chr11/116490689-116490759
Ppp1r2	U6atac-related	
Rps20	U54-related	
Tomm20	ACA14a;ACA14b	
Hist1h2bc	Histone3	
Lyzl4	mir-544	
2310037I24Rik	ACA2a;ACA2b;ACA34	
Whrn	mir-1302	
Cpne4	mir-340	mm8chr9/104891764-104891932
D1Bwg1363e	mir-671	
Ubap2l	ACA58	
Josd3	ACA1;ACA18;ACA32;	mm8chr9/15067056-15067194; mm8chr9/15066882-15066976;
	SNORA8;SNORD5;SNORD6	mm8chr9/15064196-15064310; mm8chr9/15062989-15063046
	SNORA40;SNORA25;	
Plekhj1	mir-1227	
BC005537	ACA32	
Sep15	ACA2b;SECIS	mm8chr3/144534889-144535065
Adcy3	hsa-mir-208a	
Tsr1	SNORD91	
Grik4	GRIK4_3p_UTR	mm8chr9/42271752-42271786
Hlcs	mir-692	
Sec63	RNaseP_nuc;RNaseP_RNA	
Peg10	RF_site4	mm8chr6/4705305-4705398
Ell2	ACA67;ACA67B;SNORA42	
Pank3	mmu-mir-103-1;mir-107	
Arhgef11	mir-765	
Xist	Xist	mm8chrX/99668220-99668249; mm8chrX/99668083-99668145
Hmbox1	mir-544	
1700021K19Rik	mir-922	
Grlf1	hsa-let-7e;hsa-mir-99b	mm8chr7/15655923-15655955
Tnrc6a	U6	
6230410P16Rik	hsa-let-7g;mir-135	mm8chr9/106013147-106013216; mm8chr9/106012241-106012297
Smcr7l	hsa-mir-658	mm8chr15/80077412-80077451
Nars2	mir-684	
Hist1h3f	Histone3	mm8chr13/23552317-23552415
Ntrk2	IRES_TrkB	mm8chr13/58818172-58818224

Table S3: Spatial correlated expression with $\rho \geq 0.9$ of structured UTR probes and transcripts coding for RNA binding proteins. *Neurod1*, *Prpf31*, *Tnfrsf12a*, *Ssbp4*, *Ppfia1*, *Rsf1*, *Rc3h1*, *Cnksr2*, *Prpf38a*, *Ppp1r9a*, *Lcp2*, *Cnksr3*, *Zc3hav1*, *Sfrs1*, *Samd4b*, *Prpf38b* and *Lancl1* code for an RNA binding protein and have a structured UTR. The plane orientation of all examples is the same for both transcripts. Brain region abbreviations: pallium, PAL; thalamus, TH; interbrain, IB; Hippocampal region, HIP; medulla, MY; Brain stem, BS; hypothalamus, HY; pons, P; Retrohippocampal region, RHP; Cerebellum, CB; Cerebral nuclei, CNU; olfactory bulb, OLF; Hindbrain, HB.

RNA binding protein	UTR structure	Domain	Plane	ρ
Prpf31	Degs2	PAL	sagittal	0.97
Prpf31	Scara5,Igf2,Ddo,Bspry	PAL	sagittal	0.92
Prpf31	Pde6a	PAL	sagittal	0.91
Prpf31	4732473B16Rik	PAL	sagittal	0.90
Nufip1	Prpf31	PAL	sagittal	0.90
Zc3h8	6720467C03Rik	TH	sagittal	0.96
Neurod1	Scube1	IB,TH	coronal	0.96
Neurod1	mCG1027775.1	TH	sagittal	0.96
Neurod1	mCG1027775.1	IB	sagittal	0.95
Neurod1	Kcnd2	TH	coronal	0.94
Neurod1	Vav2,Prkar2a	IB,TH	coronal	0.93
Neurod1	Rreb1	HIP	coronal	0.93
Neurod1	Kcnd2	IB	coronal	0.93
Neurod1	Pou4f1	TH,IB	coronal	0.92
Neurod1	Lhfpl2,Adcyap1r1	HIP	coronal	0.91
Neurod1	C130038G02Rik	TH	coronal	0.91
Neurod1	Sipa1l2,1810073G14Rik	HIP	coronal	0.90
Neurod1	Ebf4	TH	coronal	0.90
Tnfrsf12a	Tera	MY	coronal	0.95
Rbpms	Ppig	IB,TH	sagittal	0.95
Rbpms	Ppig	BS	sagittal	0.92
Ppie	Tom1l1,Anxa4	PAL	sagittal	0.95
Ppie	Polr2d	PAL	sagittal	0.93
Sf4	Galr2	MY	sagittal	0.94
Tnfrsf19	Slc35e4	HY	sagittal	0.93
Tnfrsf19	Flt3l	P	sagittal	0.93
Ssbp4	Tspyl2	HIP	coronal	0.93
Ssbp4	Gpd1	HIP	coronal	0.90
Ppfia1	Cd40	RHP	sagittal	0.93
Ppfia1	2700078K21Rik	RHP	sagittal	0.92
Ppfia1	Zbtb7b	RHP	sagittal	0.91
Tnfrsf10b	Ppfia1,Cd40,2610304G08Rik	RHP	sagittal	0.90
Pabpc2	Tsga14	P	sagittal	0.93
Lsm4	Stk24	IB,TH	coronal	0.93
Igf2bp2	Spryd4	HY	sagittal	0.93
Synj2	Rab37	IB	sagittal	0.92
Synj2	Sptlc2	IB	coronal	0.91
Synj2	Apbb2	IB	sagittal	0.91
Rsf1	Stk24	IB,TH	coronal	0.92
Rc3h1	Stk24	IB,TH	coronal	0.92
Rassf1	D930048N14Rik	PAL	sagittal	0.92
Polr2g	P2rx1	PAL	sagittal	0.92
Cnksr2	1110007L15Rik	TH	sagittal	0.92
Cnksr2	Ipmk	TH	sagittal	0.90
Samhd1	Stk24	IB,TH	coronal	0.91
Rnpc3	Arf6	RHP	sagittal	0.91
Prpf38a	Ppfia2	CB	sagittal	0.91
Prpf38a	Myl4	PAL	coronal	0.91

Table S3: continued

RNA binding protein	UTR structure	Domain	Plane	ρ
Ppfia2	Prpf38a	CB	sagittal	0.91
Ppp1r9a	Ngef	CNU	coronal	0.91
Myef2	Stk24	IB,TH	coronal	0.91
Lcp2	Adra2b	PAL	sagittal	0.91
Lancl2	Dlx1	RHP	sagittal	0.91
Hnrpll	Wnt6	HY	coronal	0.91
Fancl	Ddx52	HY	sagittal	0.91
Cnksr3	Slc25a37	IB	sagittal	0.91
B230120H23Rik	Nat6	RHP	sagittal	0.91
B230120H23Rik	Grb7,Gbp6	RHP	sagittal	0.90
Anks3	Cdk4	PAL	sagittal	0.91
Zc3hav1	Wars2	RHP	sagittal	0.90
Sfrs1	1200013B08Rik	OLF	sagittal	0.90
Samd4b	Fancg	RHP	sagittal	0.90
Prpf39	Siglecg	RHP	sagittal	0.90
Prpf38b	Cnnm1	HIP	coronal	0.90
Nip7	Galr2	HB	sagittal	0.90
Lancl1	Cdk4	PAL	sagittal	0.90

Table S4: Brain-wide correlated expression with $\rho \geq 0.85$ of structured UTR probes and transcripts coding for RNA binding proteins. *Khdrbs3*, *Lancl1*, *Neurod1* and *Ppp1r9a* code for an RNA binding protein and have a structured UTR. The plane orientation of all examples is the same for both transcripts.

RNA binding protein	UTR structure	Domain	Plane	ρ
Khdrbs3	Opcml	Brain	coronal	0.86
Lancl1	6530418L21Rik	Brain	coronal	0.86
Lancl1	Chn1	Brain	coronal	0.85
Neurod1	Atp2a3	Brain	sagittal	0.85
Neurod1	Chn2	Brain	sagittal	0.88
Neurod1	Trim62	Brain	sagittal	0.90
Neurod1	Zfhx2as	Brain	sagittal	0.86
Ppp1r9a	Ngef	Brain	coronal	0.86
Ppp1r9a	Phactr1	Brain	coronal	0.86
Tnrc4	Camkv	Brain	coronal	0.87
Zfp365	6530418L21Rik	Brain	coronal	0.86
Zfp365	A230097P14Rik*	Brain	coronal	0.86
Zfp365	Chn1	Brain	coronal	0.87
Zfp365	Pcsk2	Brain	coronal	0.86

Table S5: For 21 RNA binding proteins we found binding sites close to UTR structures. The relative position of the binding site to the UTR structure can be 5' or 3' (50 nts up- or downstream), in a stem, hairpin loop, interior loop (including bulges and multiloops), hinge (bifurcation), or "fiftyfifty" if exactly 50% of positions are base paired. The hits of a binding protein are quantitative significant (*) if their number is larger than the 95 percent confidence interval (p>0.95) of the number of hits in 100 dinucleotide shuffled sets of all structured UTRs.

RNA binding protein	5'	3'	stem	loop	interior	hinge	fiftyfifty	Total	p>0.95
Rbmx	7629	7490	4397	1869	1438	687	2372	25882	*
Mbnl1	6234	6300	4259	1318	913	552	2127	21703	*
Eif4b	5578	5974	3209	1403	1080	584	1986	19814	*
Pabpc1	4692	5085	1072	1429	1285	949	-	14512	*
Khsrp	4023	3951	2536	903	699	328	1070	13510	*
Rbmy1a1	1052	1114	460	346	292	147	-	3411	
Khdrbs3	349	380	90	158	90	69	48	1184	*
sap-49	332	316	202	77	53	21	74	1075	
Ybx2-a	231	234	173	97	76	38	42	891	*
Hnrnpa1	224	235	150	31	30	14	76	760	
Ybx1	130	156	66	31	28	18	39	468	
Snrpa	108	112	78	24	22	18	-	362	
Elavl2	122	73	42	29	40	8	-	314	*
Zfp36	48	60	32	22	10	8	-	180	*
Qk	66	33	6	18	10	3	-	136	
Vts1	18	25	43	9	5	-	-	100	
Sfrs2	5	7	1	-	-	-	-	13	
Sfrs1	1	1	-	-	-	-	-	2	
Elavl1	1	-	-	-	-	-	-	1	
Igf2bp1	-	1	-	-	-	-	-	1	
Rna15	1	-	-	-	-	-	-	1	

Table S6: The 4 structured putative ncRNA probes with their brain-wide correlated expressed transcripts ($\rho > 0.8$). Plane orientation is the same for both transcripts.

Image Series	Gene Symbol	Image Series	Gene Symbol	Domain	Plane	ρ
70795853	TC1462951	69353203	R3hdm2	Brain	sagittal	0.8287
70795853	TC1462951	79669509	Kcnb1	Brain	sagittal	0.8247
70795853	TC1462951	70795841	Cacnb4	Brain	sagittal	0.8125
70795853	TC1462951	69014848	Nlk	Brain	sagittal	0.8056
70795853	TC1462951	70546006	Ier5	Brain	sagittal	0.8053
74512028	mCG145872	79567505	Mef2c	Brain	coronal	0.8238
74512028	mCG145872	74658082	Itgav	Brain	coronal	0.8044
69446893	Raph1	69528040	Baalc	Brain	sagittal	0.8079
72340194	A230057G18Rik	74882787	Ptprs	Brain	coronal	0.8413
72340194	A230057G18Rik	74583118	Cri1	Brain	coronal	0.8317
72340194	A230057G18Rik	74511805	Flot2	Brain	coronal	0.8243
72340194	A230057G18Rik	74882810	Rpl15	Brain	coronal	0.8076
72340194	A230057G18Rik	74800953	Stard7	Brain	coronal	0.8009
72340194	A230057G18Rik	75042254	Glg1	Brain	coronal	0.8003

Table S7: The 33 structured putative ncRNA transcripts with locally correlated expressed transcripts ($\rho > 0.9$). The plane orientation of all examples is the same for both transcripts. Brain region abbreviations: cortical plate, CTXpl; olfactory bulb, OLF; hippocampus, HPF; striatum, STR; pallium, PAL; thalamus, TH; midbrain, MB; medulla, MY; hypothalamus, HY; pons, P; Retrohippocampal region, RHP; Hippocampal region, HIP; Hindbrain, HB; Brain stem, BS; Interbrain, IB; Cerebral nuclei, CNU.

Image Series	Gene Symbol	Image Series	Gene Symbol	Domain	Plane	ρ
74509470	D130051D11Rik	74635192	Cuzd1	P	sagittal	0.9151
70228573	A630076J17Rik	68797846	Acoxl	TH	sagittal	0.9274
69514476	A930033H14Rik	68563116	Cd4	RHP	sagittal	0.9054
71809019	9130227L01Rik	71492904	1700126L10Rik	OLF	sagittal	0.9184
71809019	9130227L01Rik	71212416	mCG146162	OLF	sagittal	0.9126
71809019	9130227L01Rik	75861005	Klra2	OLF	sagittal	0.9110
71809019	9130227L01Rik	70725615	LOC434793	OLF	sagittal	0.9093
71809019	9130227L01Rik	71212563	mCG147505	OLF	sagittal	0.9070
71809019	9130227L01Rik	70194606	Usmg1	OLF	sagittal	0.9068
71809019	9130227L01Rik	76081839	Slmap	OLF	sagittal	0.9018
71832856	Gm816	68667866	Slfn1	HIP	sagittal	0.9048
71764401	Gm831	68632014	Pip	P	sagittal	0.9153
73818824	AI452102	71492934	2010208K18Rik	OLF	sagittal	0.9008
73818824	AI452102	633294	Sfxn1	RHP	sagittal	0.9144
74736117	LOC380685	70238728	D730002M21Rik	PAL	sagittal	0.9229
71570762	Gm933	77790736	2210020M01Rik	HY	sagittal	0.9876
71570762	Gm933	68498717	Wdr78	HY	sagittal	0.9326
71570762	Gm933	73870623	C530030P08Rik*	HY	sagittal	0.9151
70238770	D830035M03Rik	68797846	Acoxl	TH	sagittal	0.9365
77924420	Gm1715	76081920	Vkorc1l1	HB	sagittal	0.9240
77924420	Gm1715	77910816	3110040N11Rik	HB	sagittal	0.9227
77924420	Gm1715	77910816	3110040N11Rik	MY	sagittal	0.9285
77924420	Gm1715	76081920	Vkorc1l1	MY	sagittal	0.9248
77924420	Gm1715	76081920	Vkorc1l1	BS	sagittal	0.9177
77924420	Gm1715	77910816	3110040N11Rik	BS	sagittal	0.9036
70300019	Gm1716	68797846	Acoxl	MB	sagittal	0.9654
74514276	LOC432876	631713	Emg1	HIP	sagittal	0.9460
74514276	LOC432876	631713	Emg1	HPF	sagittal	0.9460
74509554	LOC432907	69608088	Crygc	OLF	sagittal	0.9113
74509554	LOC432907	69608088	Crygc	CTXpl	sagittal	0.9052
70723050	LOC433280	69261398	BC026439	P	sagittal	0.9016
70723223	LOC433405	68151448	Tsga14	P	sagittal	0.9050
70723223	LOC433405	70723632	1700022A21Rik	PAL	sagittal	0.9014
70303725	LOC433503	79762714	Gpx3	HIP	sagittal	0.9186
70303725	LOC433503	79762714	Gpx3	HPF	sagittal	0.9171
74635186	LOC433965	69608088	Crygc	OLF	sagittal	0.9091
73635375	mCG1030139	69258178	Mtnr1b	IB	sagittal	0.9068
73635375	mCG1030139	69258178	Mtnr1b	TH	sagittal	0.9157
74635445	mCG1044749.1	70228167	9530002K18Rik	OLF	sagittal	0.9370
74635445	mCG1044749.1	70786839	LOC433816	OLF	sagittal	0.9331
74635445	mCG1044749.1	71212578	mCG147545	OLF	sagittal	0.9299
74635445	mCG1044749.1	77454548	Xlr3a	OLF	sagittal	0.9276
74635445	mCG1044749.1	73748776	F830225E14Rik*	OLF	sagittal	0.9269
74635445	mCG1044749.1	68196921	Igsf9	OLF	sagittal	0.9246
74635445	mCG1044749.1	75988299	BC028799	OLF	sagittal	0.9148
74635445	mCG1044749.1	74431453	Slc16a8	OLF	sagittal	0.9147
74635445	mCG1044749.1	71574600	C79127	OLF	sagittal	0.9134
74635445	mCG1044749.1	69608088	Crygc	OLF	sagittal	0.9120

Table S7: continued

Image Series	Gene Symbol	Image Series	Gene Symbol	Domain	Plane	ρ
74635445	mCG1044749.1	74821969	Fabp4	OLF	sagittal	0.9114
74635445	mCG1044749.1	75831767	Cd300lf	OLF	sagittal	0.9114
74635445	mCG1044749.1	70725579	Gm1966	OLF	sagittal	0.9104
74635445	mCG1044749.1	71808741	4930451G09Rik	OLF	sagittal	0.9018
74635445	mCG1044749.1	68151448	Tsga14	P	sagittal	0.9258
74635445	mCG1044749.1	70228167	9530002K18Rik	CTXpl	sagittal	0.9158
74635445	mCG1044749.1	73748776	F830225E14Rik*	CTXpl	sagittal	0.9067
74635445	mCG1044749.1	70786839	LOC433816	CTXpl	sagittal	0.9056
74635445	mCG1044749.1	68196921	Igsf9	CTXpl	sagittal	0.9046
74635445	mCG1044749.1	77454548	Xlr3a	CTXpl	sagittal	0.9022
74510254	mCG1048826	631713	Emg1	HIP	sagittal	0.9302
74510254	mCG1048826	631713	Emg1	HPF	sagittal	0.9302
71212268	mCG145872	69257660	Gpr87	HY	sagittal	0.9098
71212268	mCG145872	68861730	2810410C14Rik	HY	sagittal	0.9008
74736456	LOC546269	73513492	AY702103	MY	sagittal	0.9086
73513717	E130008D07Rik	68797846	Acoxl	TH	sagittal	0.9129
75043641	LOC545466	70946226	Cybasc3	HB	sagittal	0.9338
75043641	LOC545466	70946226	Cybasc3	MY	sagittal	0.9403
75043641	LOC545466	71609044	Zfp54	MY	sagittal	0.9101
75043641	LOC545466	74277702	LOC545904	MY	sagittal	0.9030
75043641	LOC545466	73927700	7420458B01Rik*	MY	sagittal	0.9028
75043641	LOC545466	70946226	Cybasc3	BS	sagittal	0.9004
73808450	9530020O07Rik*	68845741	Frap1	HY	sagittal	0.9315
74427061	0610007N19Rik	74300729	C330006D17Rik*	P	sagittal	0.9002
74427061	0610007N19Rik	74580978	TC1480793	HY	sagittal	0.9442
74427061	0610007N19Rik	73767187	F730219E24Rik*	HY	sagittal	0.9360
74427061	0610007N19Rik	69524566	AI461788	HY	sagittal	0.9232
74427061	0610007N19Rik	74357833	1700026J04Rik*	HY	sagittal	0.9071
74635108	1700123J19Rik	68797846	Acoxl	MB	sagittal	0.9729
69443268	5031434O11Rik	71891539	Hoxa7	PAL	sagittal	0.9009
70218318	4930545L08Rik	71148223	Mm.152121	HY	sagittal	0.9336
70218318	4930545L08Rik	70302890	Ugt1a1	HY	sagittal	0.9183
70295536	Gm57	68797846	Acoxl	TH	sagittal	0.9115
70295859	Gm559	69028635	0610012H03Rik	CNU	sagittal	0.9375
70295859	Gm559	69028635	0610012H03Rik	STR	sagittal	0.9384
70295948	Gm582	79534912	Aqp1	RHP	sagittal	0.9027

Table S8: For correlated expressed ncRNAs we predict putative RNA-RNA interaction sites to their correlated RNA using `RNAplfold` and `RNAplex`. GC content and length of the interaction site are the average of both sequences. The RNA-RNA interaction is shown in dot-bracket format in which the symbol '&' separates both sequences. Abbreviation: Minimum free energy, MFE. The plane orientation of all examples is the same for both transcripts. Brain region abbreviations: cortical plate, CTXpl; olfactory bulb, OLF; hypothalamus, HY; pons, P; Retrohippocampal region, RHP.

ncRNA	mRNA	interaction	MFE	GC	length	p-value	Domain	Plane	ρ
LOC433503	Gpx3 (3'-UTR)	(((((.(((((((((((((&))))))))))))))))))	-22.80	0.57	19	0.0097	P	sagittal	0.90
TC1462951	Kcnb1 (3'-UTR)	((((((((((&))))))))))	-14.34	0.65	10	0.1421	Brain	sagittal	0.82
TC1462951	Nlk (3'-UTR)	((((((((((((&)))))).))))))	-13.05	0.60	12	0.2312	Brain	sagittal	0.81
TC1462951	Ier5 (3'-UTR)	((((((((.((((((&)))))).))))))))	-13.16	0.43	15	0.2335	Brain	sagittal	0.81
TC1462951	R3hdm2 (3'-UTR)	((((((((((.((((..(((((&))))))))).))))))))))	-13.13	0.45	22	0.2912	Brain	sagittal	0.83
TC1462951	Dusp3 (3'-UTR)	(((.((((((((((((&))))))))))))..)))	-13.56	0.70	16	0.3209	Brain	sagittal	0.85
A930033H14Rik	Cd4 (5'-UTR)	(((((((((..(((.((((...(((((&)))))...))))..)))..)))).))).))	-15.18	0.53	27	0.2256	RHP	sagittal	0.91
mCG145872	Itgav (3'-UTR)	(((((((((&)))))))))	-12.91	0.67	9	0.2700	Brain	coronal	0.80
LOC432907	Crygc (3'-UTR)	((((.(((((((&))))))).))))	-10.73	0.58	12	0.6508	OLF	sagittal	0.91
LOC432907	Crygc (3'-UTR)	((((.(((((((&))))))).))))	-10.73	0.58	12	0.6508	CTXpl	sagittal	0.91
LOC433280	BC026439 (5'-UTR)	(((((.(((((((((.((.((((&)))).)).)))))))))........)))))	-11.58	0.62	30	0.6652	P	sagittal	0.90
9530020O07Rik*	Frap1 (3'-UTR)	((((((.(((((&)))))))))))	-10.67	0.79	12	0.7205	HY	sagittal	0.93
D130051D11Rik	Cuzd1 (3'-UTR)	((((.((((.((((&)))).)))).))))	-8.00	0.50	14	0.9425	P	sagittal	0.92

Additional Figures

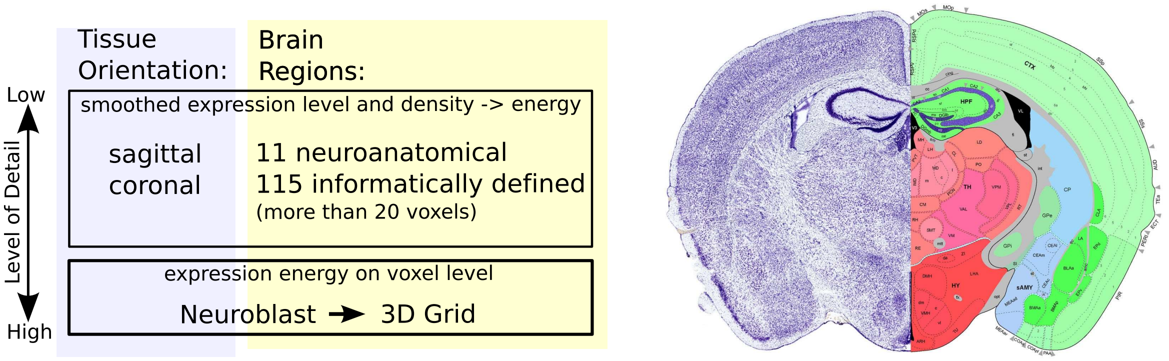

Figure S1: Mapping Allen Mouse Brain Atlas expression data. Tissue sections are generated in either the sagittal or coronal plane. The coronal plane contains both hemispheres and thus provides a template for more accurate registration and informatically defined structures. The measured expression level and density are combined to create expression energy. Both planes are mapped to 11 large neuroanatomical regions and 115 informatically defined regions. The image on the right shows a Nissl stain and the annotated regions of a coronal tissue section from the Allen Mouse Brain Atlas. Each informatically defined domain consists of more than 20 voxels that are interpolated over all images in either the sagittal or coronal orientation to a 3-dimensional grid. The 3D grid data represents the highest level of detail and is used for correlated expression analyses by NeuroBlast.

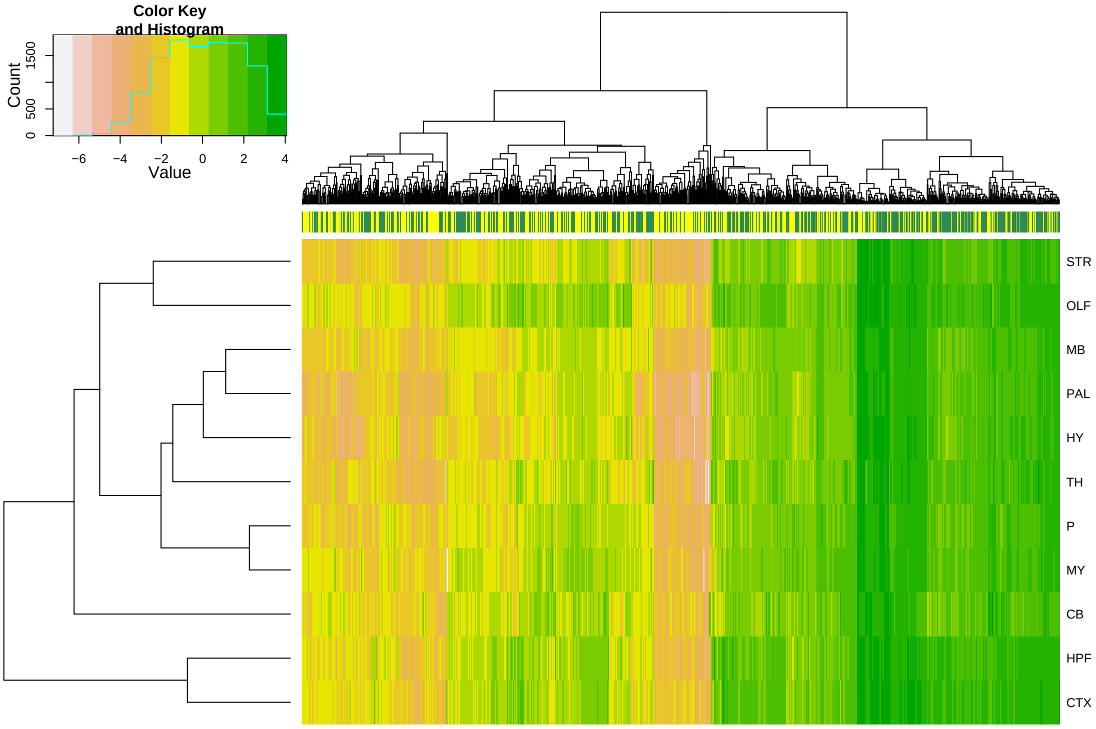

Figure S2: Heatmap of expression energy log-z-scores normalized over UTR probes and brain regions. The 5,126 structured UTR probes (green lines in the barcode in the top) and 4,467 non-structured UTR probes (yellow) are hierarchical clustered by their energy distribution (tree in the top) in the 11 neuroanatomical regions which are also hierarchical clustered (tree on the left side). The right branch of the probe expression tree exhibiting large expression is highly dominated by structured UTR probes. A random sample of 1/10th of all data was used. Brain region abbreviations: cortex, CTX; olfactory bulb, OLF; hippocampus, HPF; striatum, STR; pallium, PAL; thalamus, TH; midbrain, MB; medulla, MY; hypothalamus, HY; cerebellum, CB; pons, P.

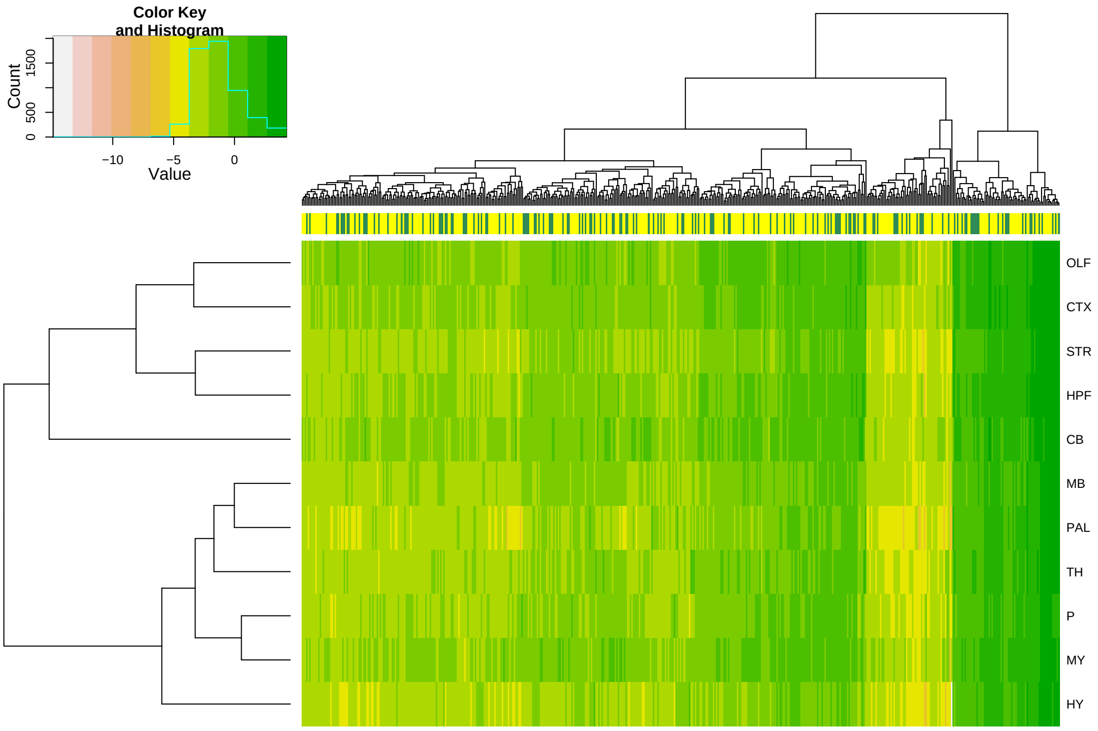

Figure S3: Heatmap of expression energy log-z-scores normalized over ncRNA probes and brain regions. The 163 structured putative ncRNA probes (green lines in the barcode in the top) and 338 non-structured ncRNA probes (yellow) are hierarchical clustered by their energy distribution (tree in the top) in the 11 neuroanatomical regions which are also hierarchical clustered (tree on the left side). Structured and non-structured putative ncRNAs have relatively similar distribution in the hierarchical expression tree. Brain region abbreviations: cortex, CTX; olfactory bulb, OLF; hippocampus, HPF; striatum, STR; pallium, PAL; thalamus, TH; midbrain, MB; medulla, MY; hypothalamus, HY; cerebellum, CB; pons, P.

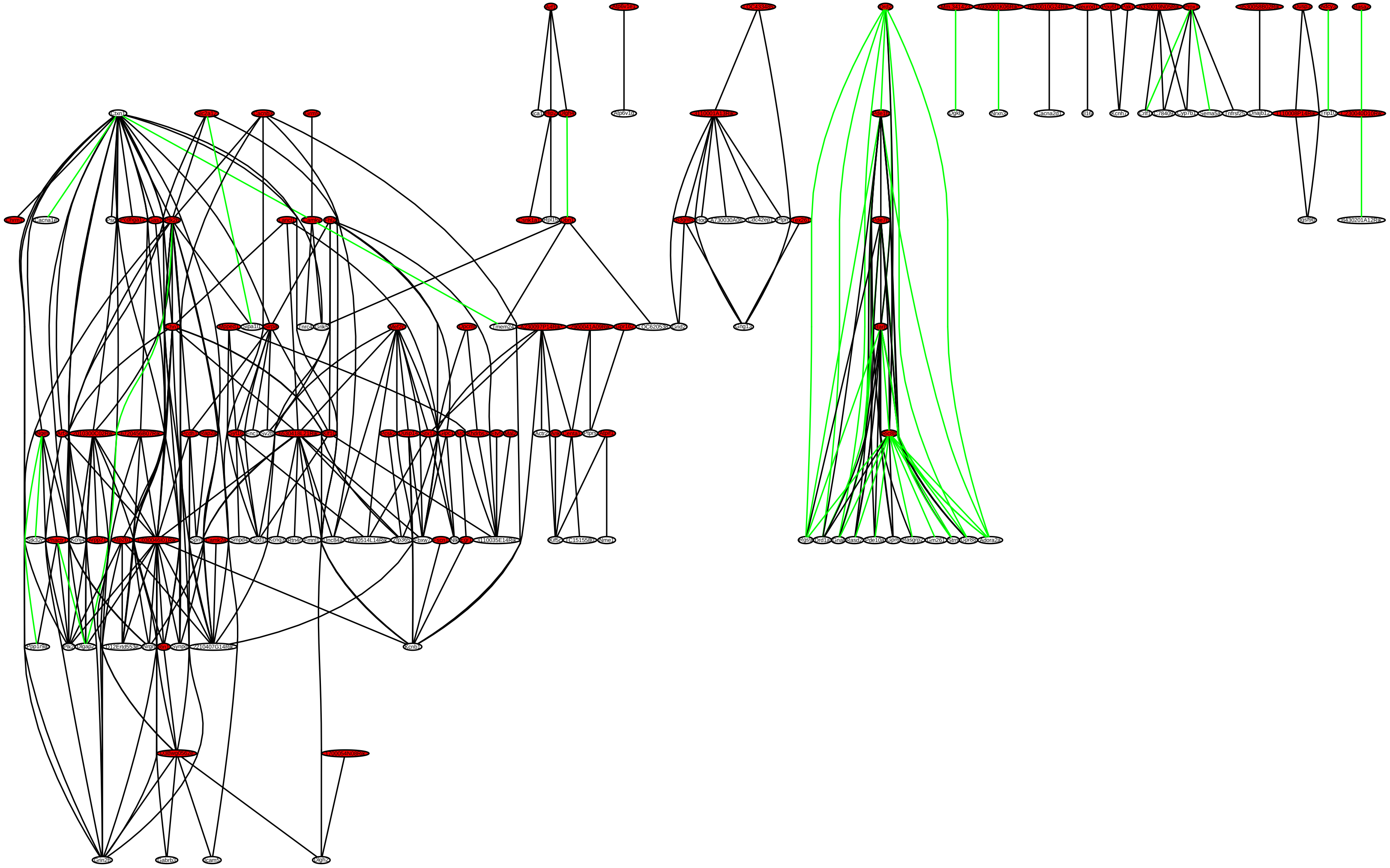

Figure S4: The correlated expression network of 78 structured Allen Mouse Brain Atlas transcripts (red nodes) with correlated expression over the entire brain. They are involved in 352 correlation pairs (edges, $\rho \geq 0.85$). Some of them (green edges) also have spatial expression enrichments ($\rho \geq 0.9$).

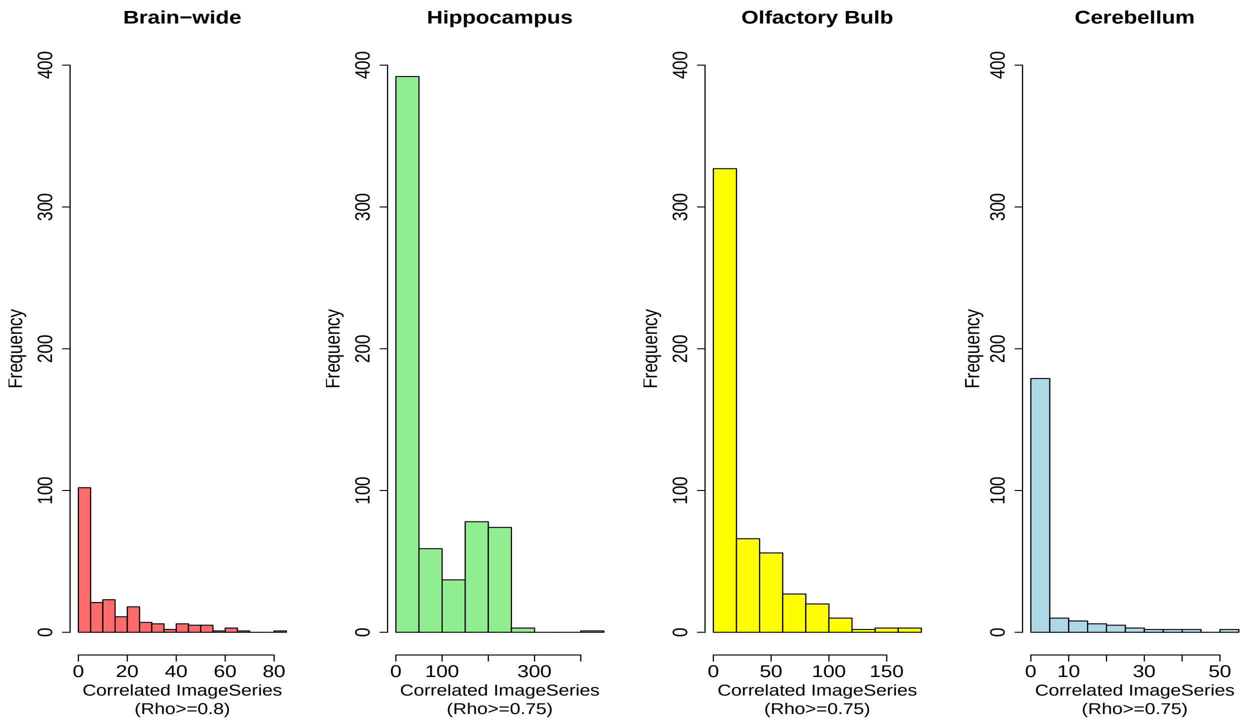

Figure S5: The number of correlated Allen Mouse Brain Atlas riboprobes per structured probe. Correlated coronal image series are counted brain-wide ($\rho \geq 0.8$), and in the brain regions hippocampus ($\rho \geq 0.75$), olfactory bulb ($\rho \geq 0.75$) and cerebellum ($\rho \geq 0.75$) (from left to right). In the 2,926 brain-wide correlation pairs (left barplot) 212 structured UTR transcripts are involved, of which 89 have less than 5 correlated expressed RNAs.

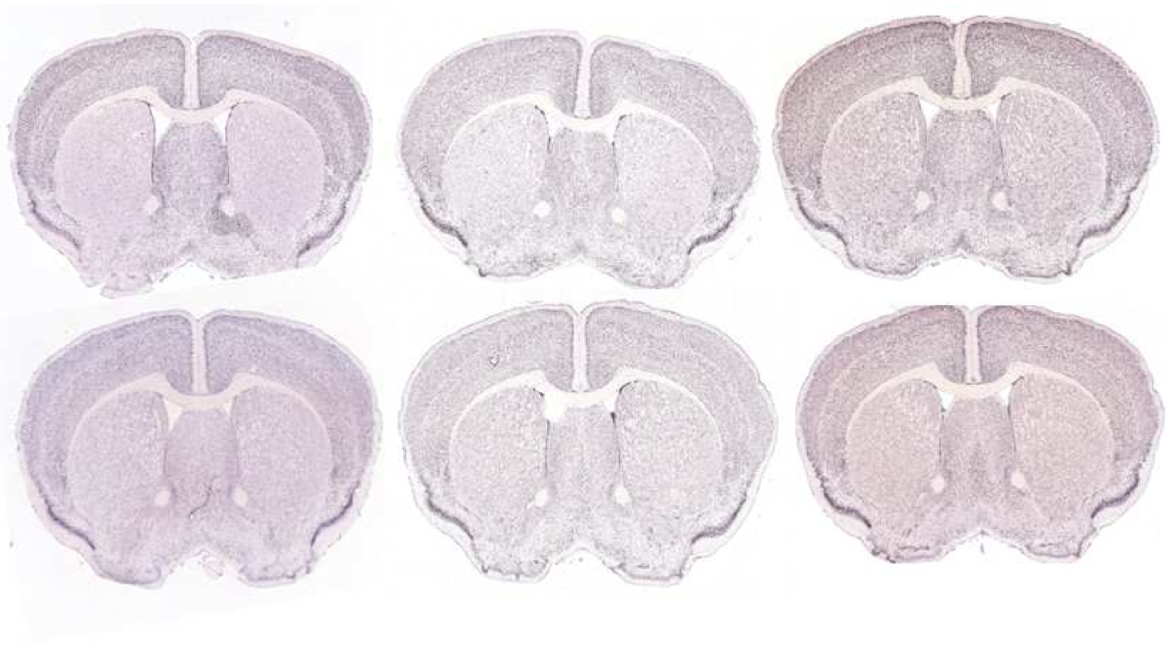

Figure S6: A representative coronal section showing *in situ* hybridization data primarily from the cortex and striatum from the structured ncRNA candidate *A230057G18Rik* (Miat) and the correlated expressed transcripts *Ptprs*, *Cri1*, *Flot2*, *Rpl15*, and *Stard7* (left to right, top to bottom). All probes show higher expression in the cortex compared to the striatum.

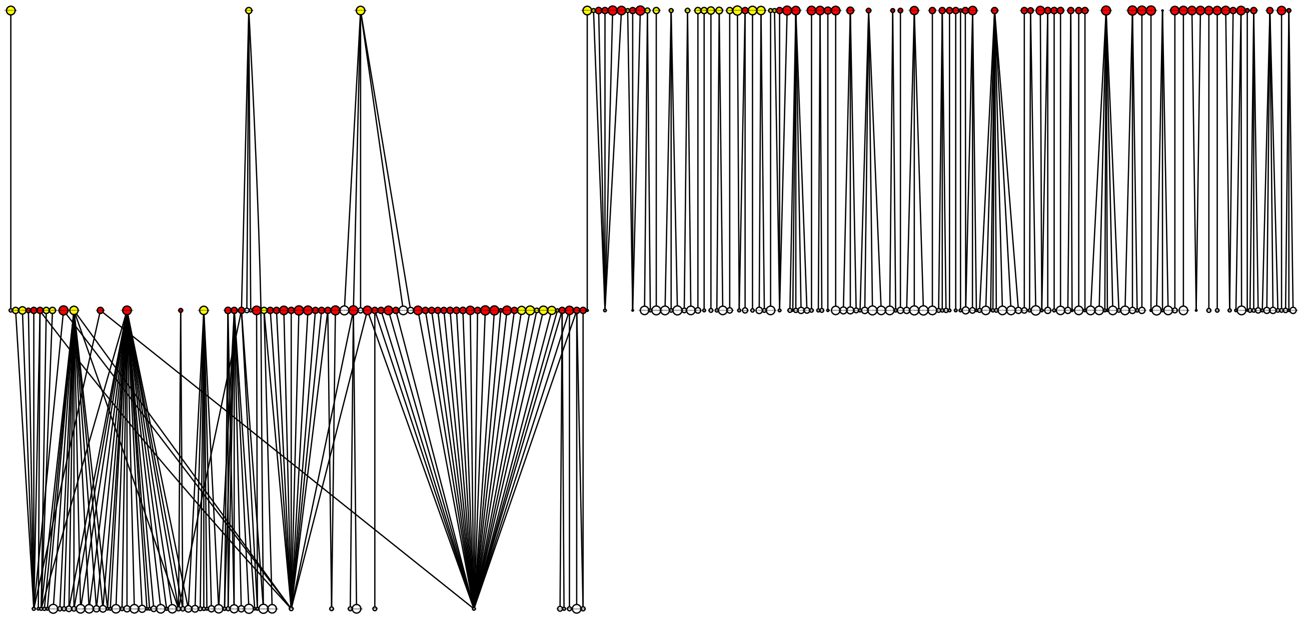

Figure S7: The correlation network of 134 putative ncRNA transcripts with strong locally correlated expression patterns. They are involved in 326 correlation pairs (edges, $\rho > 0.9$). Red nodes represent transcripts without secondary RNA structure prediction and yellow nodes with structure predictions. However, the protein-coding probes *Acoxl* (covariance to 25 putative ncRNAs in interbrain and thalamus, 7 in midbrain), *Sst* (2 in cerebellum), *Tsga14* (16 mostly in pons), and *Pip* (3 in pons) have high local correlation coefficients to several long noncoding RNAs, but their expression in these domains is too low to get meaningful correlation coefficients.

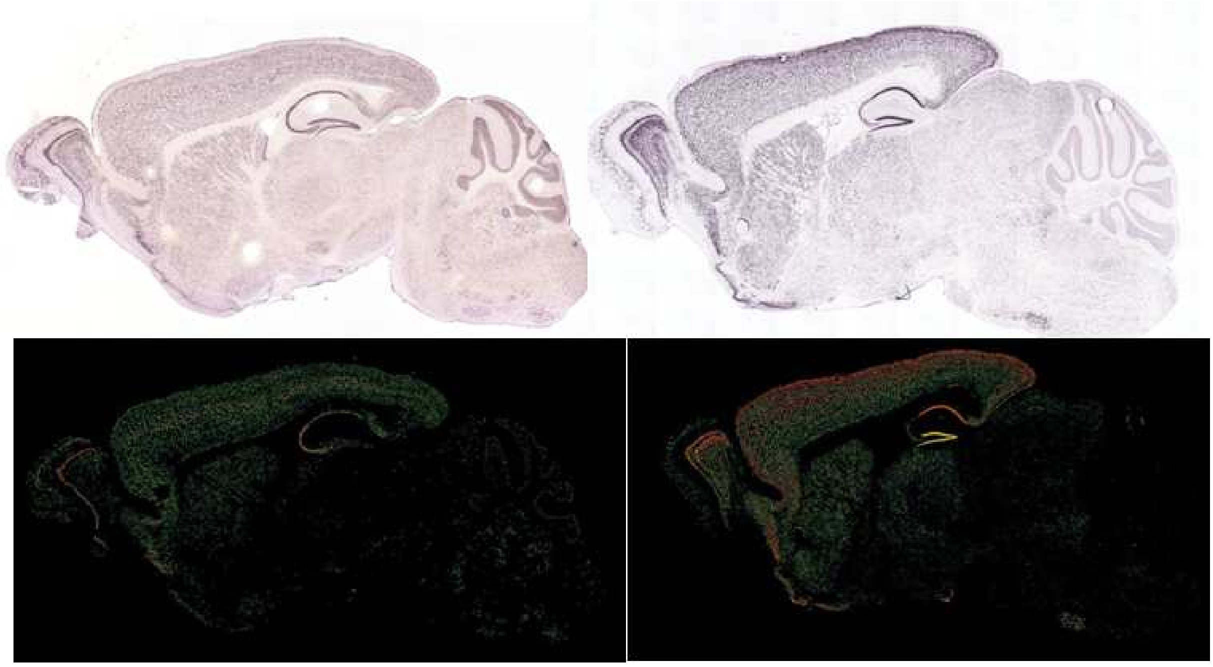

Figure S8: Gene expression shown by in situ hybridization (ISH) in one sagittal section from *TC1462951* (upper left panel) and *Kcnb1* (upper right panel). The lower panels show the corresponding expression mask for each of the ISH images. Both probes show strong expression in the cortex, hippocampus, striatum, olfactory bulb and lower expression in othe brain regions such as the pons, medulla, midbrain, and cerebellum.

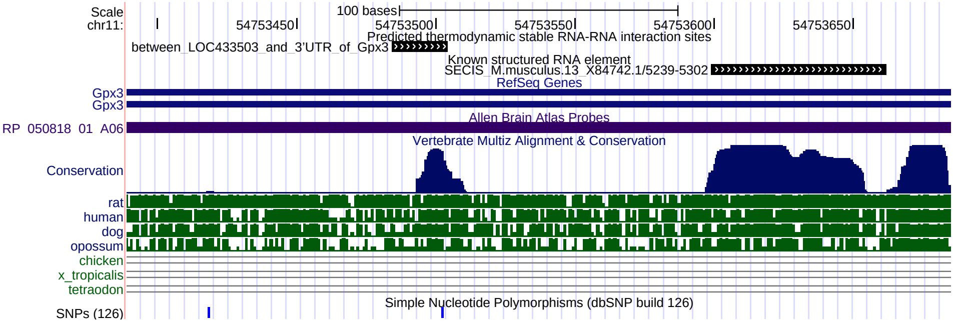

Figure S9: The predicted thermodynamic stable RNA-RNA interaction site between the structured ncRNA *LOC433503* and the 3'-UTR of *Gpx3* is located 100 nts upstream of a selenocysteine insertion sequence (SECIS) element as shown by tracks in the UCSC genome browser.

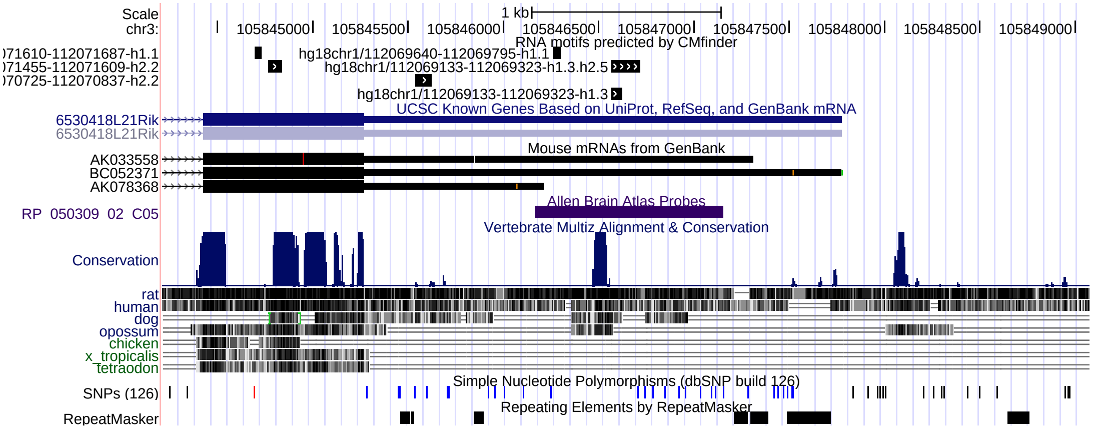

Figure S10: A large loop of the 3'-UTR structure *hg18chr1/112069133-112069323-h1.3.h2.5* of the hypothetical protein *6530418L21Rik* hosts a binding motif to the zinc finger protein *Zfp36* as shown by tracks in the UCSC genome browser. *6530418L21Rik* is brain-wide correlated expressed to the RNA binding proteins *Zfp365* and *Lancl1* .

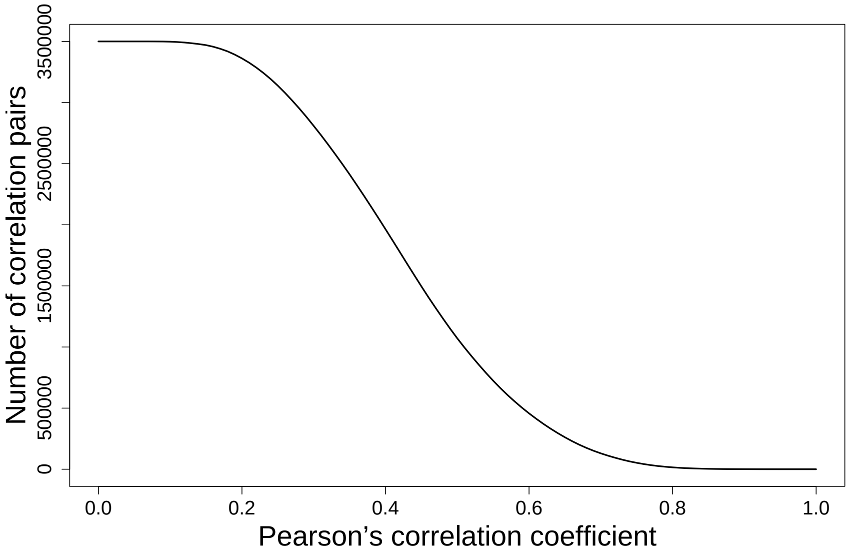

Figure S11: The relationship between the minimum Pearson's correlation coefficient ρ and the number of correlation pairs for regional covariance patterns of the 477 putative ncRNAs (intergenic and intronic RNA probes).

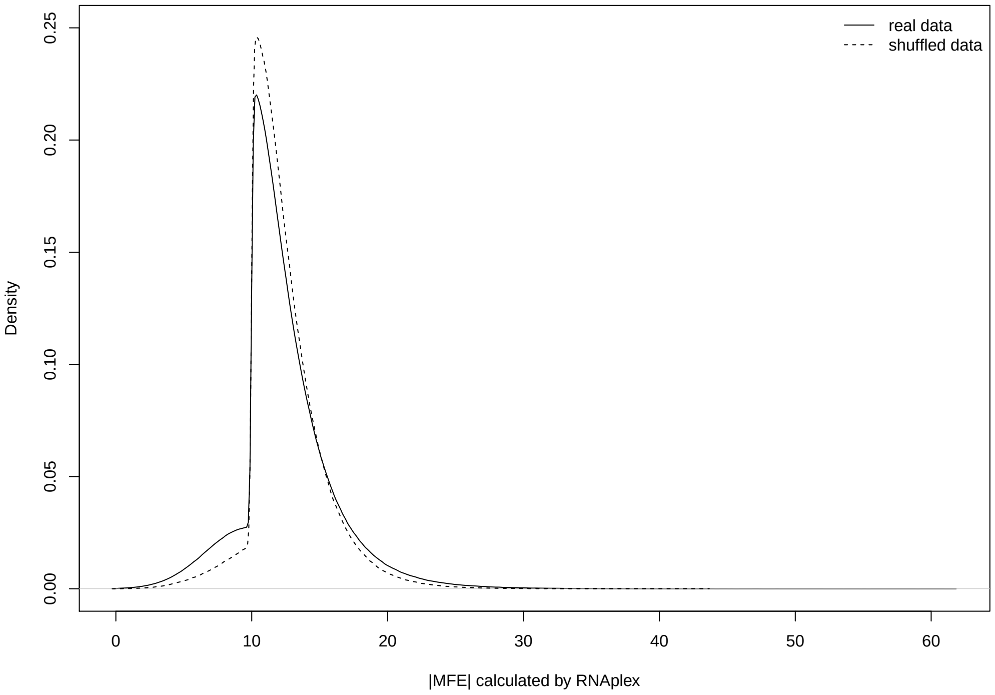

Figure S12: Extrem-value distribution of the MFE of RNA-RNA interactions for the real data and all dinucleotide shuffled data.

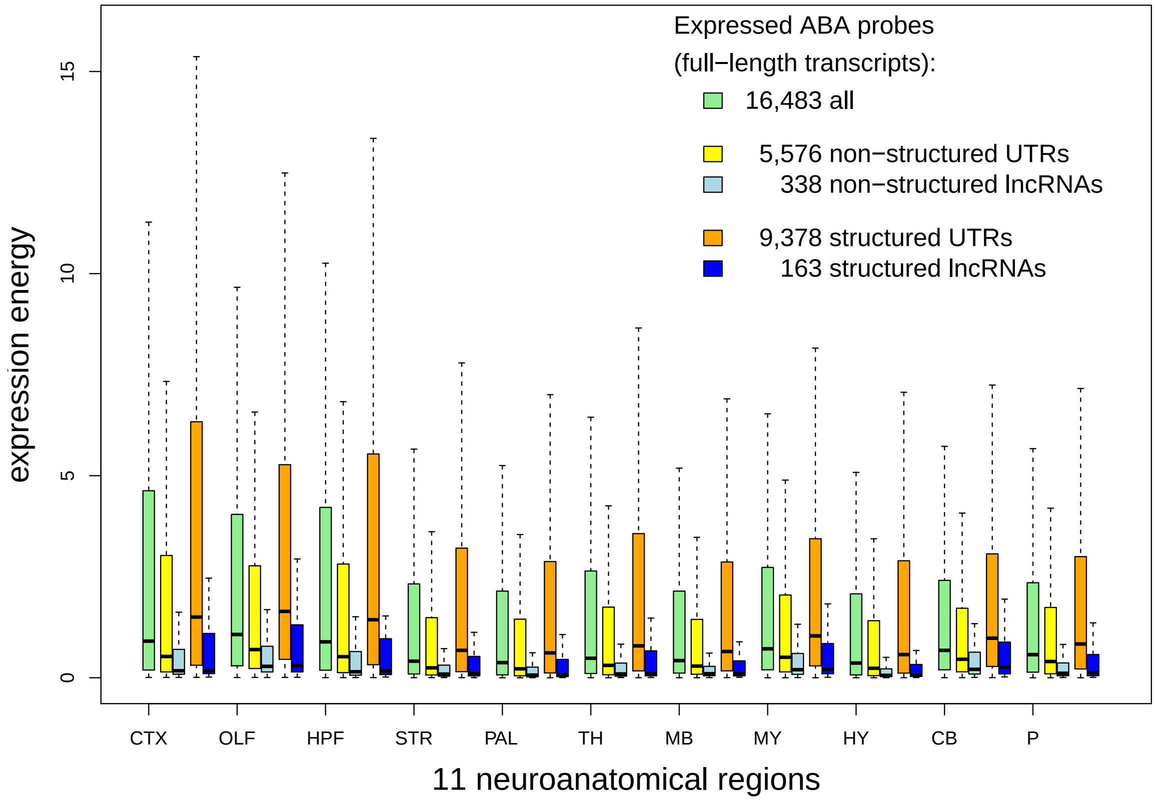

Figure S13: Comparison of expression energy distribution of all expressed Allen Mouse Brain Atlas probes, structured and nonstructured ncRNA and UTR probes in 11 neuroanatomical regions. UTR probes are considered as structured if any transcriptonal isoform has an UTR structure. Secondary RNA structures are predicted by CMfinder. The box plot shows 1.5 interquartile range (dotted line), lower and upper quartile (box), and median (thick black line in the box). Brain region abbreviations: cortex, CTX; olfactory bulb, OLF; hippocampus, HPF; striatum, STR; pallium, PAL; thalamus, TH; midbrain, MB; medulla, MY; hypothalamus, HY; cerebellum, CB; pons, P.

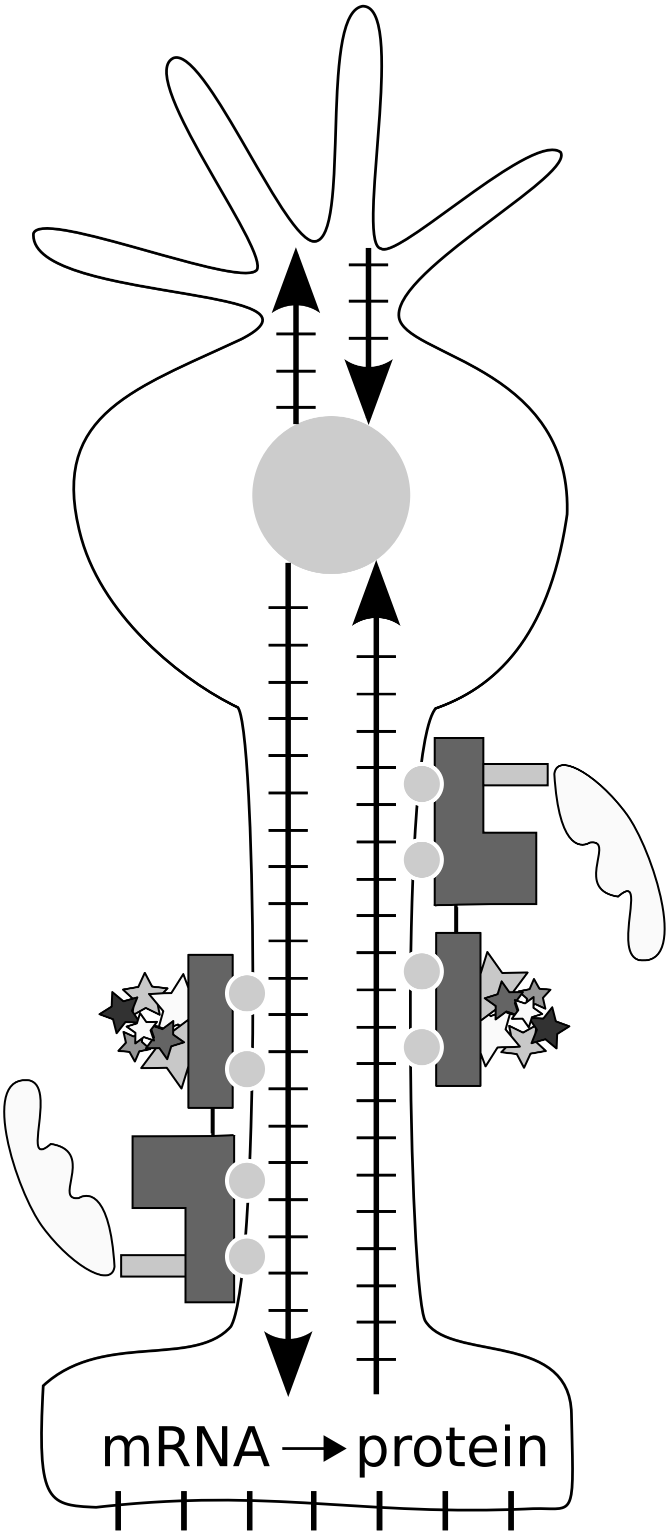

Figure S14: This cartoon illustrates the directed RNA transport theory. In a neuron consisting of dentrites, cell body with nucleus, axon and synapse (from top to bottom) the intracellular signal is transported along the cytoskeleton (symbolized by train tracks) from the nucleus to the synapse and dentrites as well as in the opposite direction back to the nucleus. The trains and railway carriages symbolize the motor and transport proteins and the stars symbolize the transported cargo. The transport is only going in one direction and, thus, the mRNAs coding for proteins involved in the signalling pathways have to be transported first to the location where the new transport starts. The UTR structures are needed to attach the mRNAs (stars), which themselves partly code for transport proteins, to the transport machinery (railway carriage).

Supplementary References

Cook, K. B., Kazan, H., Zuberi, K., Morris, Q., and Hughes, T. R. (2010). Rbpdb: a database of rna-binding specificities. *Nucleic Acids Res*.

Lenhard, B. and Wasserman, W. W. (2002). Tfbs: Computational framework for transcription factor binding site analysis. *Bioinformatics*, **18**(8), 1135–1136.

Torarinsson, E., Yao, Z., Wiklund, E. D., Bramsen, J. B., Hansen, C., Kjems, J., Tommerup, N., Ruzzo, W. L., and Gorodkin, J. (2008). Comparative genomics beyond sequence-based alignments: Rna structures in the encode regions. *Genome Res*, **18**(2), 242–251.
